# RNA hybrid-capture next-generation sequencing has high sensitivity in identifying known and less characterized oncogenic and likely oncogenic *NTRK* fusions in a real-world standard-of-care setting

**DOI:** 10.3389/fgene.2025.1550706

**Published:** 2025-05-02

**Authors:** Zachary D. Wallen, Marni Tierno, Erica Schnettler, Alison Roos, Michelle Green, Kobina Amoah, Rebecca A. Previs, Stephanie Hastings, Sarabjot Pabla, Taylor J. Jensen, Brian Caveney, Marcia Eisenberg, Pratheesh Sathyan, Shakti H. Ramkissoon, Eric A. Severson

**Affiliations:** ^1^ Labcorp, Durham, NC, United States; ^2^ Illumina, San Diego, CA, United States; ^3^ Labcorp, Burlington, NC, United States; ^4^ Wake Forest Comprehensive Cancer Center, Wake Forest School of Medicine, Department of Pathology, Winston-Salem, NC, United States

**Keywords:** *NTRK*, fusions, clinical utility, targeted therapy, genomics, next-generation sequencing, diagnostics, oncology

## Abstract

**Introduction:**

*NTRK1*, *NTRK2*, and *NTRK3* gene fusions are rare oncogenic driver alterations found in diverse tumor types of adults and children. They are clinically important biomarkers as tumors harboring these genomic alterations have high response rates to targeted therapy. Routine testing for *NTRK* fusions and treatment with TRK inhibitors has been recommended in multiple tumor types; however, differences between testing technologies used for detecting *NTRK* fusions can result in variable likelihoods of identification.

**Methods:**

To assess the prevalence of *NTRK* fusions in a real-world standard-of-care setting, we analyzed data from 19,591 FFPE samples encompassing 35 solid tumor types submitted for comprehensive genomic profiling (CGP) as part of routine clinical care. CGP testing included DNA hybrid-capture sequencing to detect small variants, copy number alterations, microsatellite instability (MSI), and tumor mutational burden (TMB). RNA hybrid-capture sequencing was concurrently performed to detect fusions and splice variants. Detected *NTRK* fusions were categorized as oncogenic, likely oncogenic, or variant of unknown significance (VUS) based on the fusion partner, orientation, and breakpoint position.

**Results:**

73 oncogenic or likely oncogenic *NTRK* fusions were identified in 69 unique tumor specimens across 19 tumor types for a total cohort prevalence of 0.35%. Tumor types with the highest *NTRK* fusion prevalence included glioblastoma (1.91%), small intestine (1.32%), and head and neck (0.95%) tumors with other solid tumor types ranging from 0.19% (uterine) to 0.63% (breast). We identified diverse intra and inter-chromosomal partner genes for *NTRK* fusions across all tumor types. Most *NTRK* fusions were detected in only one tumor specimen, though some recurrent fusions were noted with *ETV6*, *TPM3*, *LMNA*, *EML4*, *TPR*, *PEAR1*, *IRF2BP2*, and *KANK1* fusion partners. Most *NTRK* fusions were mutually exclusive from other genomic driver alterations, however, almost a third of tumor specimens (29%) contained at least one co-occurring genomic driver, which may affect treatment decisions.

**Discussion:**

The high prevalence of oncogenic and likely oncogenic *NTRK* fusions detected in our analysis suggests that RNA hybrid-capture-based sequencing for fusion detection is a highly sensitive method for identifying clinically meaningful known and novel *NTRK* fusions, which may be missed with other detection methods, directly impacting therapeutic options and patient outcomes.

## 1 Introduction

The tropomyosin receptor kinase (TRK) family of receptor tyrosine kinases are encoded by the *NTRK1, NTRK2,* and *NTRK3* (*NTRK*) genes and play a critical role in neuronal development and differentiation (Amatu et al., 2019; Cocco et al., 2018). The common ligands of TRK receptors are neurotrophins, a family of growth factors critical to the functioning of the nervous system. The activation of TRK receptors by neurotrophin leads to the activation of signal cascades, including the RAS and PI3K pathways, promoting survival and other regulatory pathways in cells. The most common mechanism of oncogenic activation of the TRK receptors is via fusions involving the *NTRK* genes (Cocco et al., 2018). *NTRK* fusions are the consequence of large-scale chromosomal events, including inversions, deletions, and translocations, and result in the fusion of the C-terminal tyrosine kinase domain (TKD) of *NTRK* with an N-terminal fusion partner (Amatu et al., 2019; Wong et al., 2020). *NTRK* genes have >60 known fusion partners across multiple tumor types and the number is growing as novel fusion partners are identified (Kummar and Lassen, 2018). *NTRK* gene fusions are found at a low frequency (<1%) across multiple common pediatric and adult solid tumor types but are enriched (>90%) in a few rare tumors (secretory breast carcinoma, mammary analogue secretory carcinoma, cellular or mixed congenital mesoblastic nephroma, and infantile fibrosarcoma) (Amatu et al., 2019; Cocco et al., 2018; Okamura et al., 2018). In-frame *NTRK* fusions result in constitutive activation of the TRK receptor and downstream signaling pathways (Amatu et al., 2019; Cocco et al., 2018). Genomic characterization of *NTRK* fusion-positive cancers has demonstrated these tumors are typically devoid of other oncogenic drivers, with the notable exception of microsatellite instability-high (MSI-H) colorectal cancer (CRC) (Westphalen et al., 2021; Gatalica et al., 2019; Wang H. et al., 2022).

Routine testing for *NTRK* fusions or treatment with TRK inhibitors has been recommended in >25 different tumor types (Klink et al., 2022). Variations in testing technologies can result in significant differences in the likelihood of fusion identification (Marchio et al., 2019). Single gene tests, hotspot panels, and inadequately baited DNA-based next-generation sequencing (NGS) have technical limitations precluding accurate detection of fusions (Penault-Llorca et al., 2019; Solomon et al., 2019). Highly sensitive RNA sequencing methods such as hybrid capture are optimal for comprehensively identifying actionable fusions, including *NTRK* fusions in solid tumors. In non-small cell lung cancer (NSCLC), the National Comprehensive Cancer Network (NCCN) Clinical Practice Guidelines in Oncology recommend biomarker testing be performed using broad NGS panels to detect druggable rearrangements or fusions involving *ALK, NTRK, ROS1*, and *RET* with consideration of RNA sequencing if not previously performed (Ettinger et al., 2022).

*NTRK* gene fusions were the first gene-specific alterations to receive US Food and Drug Administration (FDA) approval in a histology-agnostic manner across all solid tumors (Drilon, 2019). Larotrectinib (inhibitor of TRKA, TRKB, and TRKC) and entrectinib (inhibitor of TRKA, B and C, *ROS1,* and *ALK*) are both FDA-approved for adult and pediatric patients with metastatic, unresectable solid tumors harboring *NTRK* fusions that have progressed on prior therapies or when no suitable treatment is available. In phase I and II trials of larotrectinib, an objective response was observed in 79% of evaluable patients, with 16% having complete responses (Hong et al., 2020). At a median follow-up of 25.8 months, entrectinib demonstrated a complete or partial response in 61.2% of patients (Demetri et al., 2022). In 2024, the FDA granted accelerated approval to repotrectinib as the third pan-tumor approval for the treatment of adult and pediatric patients (12 years or older) with unresectable solid tumors harboring an NTRK gene fusion with locally advanced or metastatic disease after treatment progression or those with no satisfactory alternative therapy options, based on efficacy data from the phase 1/2 TRIDENT-1 trial in TRK inhibitor naïve patients and those who previously received a TRK inhibitor (Solomon et al., 2023). Entrectinib, larotrectinib, and repotrectinib are effective across pediatric and adult solid tumors and responses are independent of the *NTRK* fusion gene and fusion partner. Despite the efficacy of TRK inhibitors, acquired resistance to TRK inhibitors, including the emergence of on-target *NTRK* kinase domain mutations or off-target bypass mechanisms, remains a clinical unmet need. Second-generation TRK inhibitors are in development for on-target *NTRK* kinase domain mutations (Drilon, 2019; Cocco et al., 2019a).

The immunotherapy biomarker landscape in patients with *NTRK* fusions has been explored, and the association of *NTRK* fusions with tumor mutational burden (TMB) is discrepant between studies, except for in microsatellite instability-high (MSI-H) CRC (Gatalica et al., 2019; Wang H. et al., 2022; Rosen et al., 2020; Bazhenova et al., 2021). Programmed death-ligand 1 (PD-L1) expression has been detected in around 25% of *NTRK* fusion*-*positive cases, including MSI-H cases (Gatalica et al., 2019). NCCN guidelines emphasize that for patients with advanced NSCLC, testing for all recommended biomarkers should be performed before initiating single-agent immunotherapy due to the lack of response in tumors harboring actionable driver alterations (Ettinger et al., 2022; Mazieres et al., 2019). However, there has been minimal investigation of treatment sequencing in patients with *NTRK* fusion-positive tumors concerning targeted therapy *versus* immunotherapy and the role of immunotherapy after targeted therapy resistance.

To assess the prevalence of *NTRK* fusions in a real-world standard-of-care setting, we (Amatu et al., 2019) describe the landscape of oncogenic and likely oncogenic *NTRK* fusions detected across solid tumors, including novel fusion partners, by hybrid-capture next-generation RNA sequencing performed in a reference laboratory as part of patients’ routine clinical care and (Cocco et al., 2018) characterize the co-occurring alterations and immunotherapy-related signatures detected in *NTRK* fusion-positive solid tumors.

## 2 Methods

### 2.1 Patient cohort

Approval for this study, including waiver of informed consent, was obtained from the Western Institutional Review Board Copernicus Group (WCG protocol # 1340120).

We retrospectively analyzed comprehensive genomic profiling (CGP) and PD-L1 22C3 immunohistochemistry (IHC) data from 19,591 FFPE solid tumor samples submitted for NGS testing at a reference laboratory (OmniSeq/Labcorp, Buffalo, NY, United States) as part of patients’ routine clinical care between June 2021 and August 2024 (Supplementary Table S1).

### 2.2 Comprehensive genomic profiling (CGP)

DNA and RNA were co-extracted from FFPE tissue specimens and submitted for library preparation and sequencing using the hybrid-capture-based TruSight^®^ Oncology (TSO) 500 assay (Illumina, San Diego, CA, United States) as part of OmniSeq^®^ INSIGHT (OmniSeq/Labcorp, Buffalo, NY, United States). OmniSeq^®^ INSIGHT is a comprehensive genomic and immune profiling assay performed in a laboratory accredited by the College of American Pathologists and certified by the Clinical Laboratory Improvement Amendments (Conroy et al., 2021). Within the genomic profiling framework of the assay, DNA sequencing with hybrid capture detects small nucleotide variants (SNVs) in exonic regions of 523 genes, copy number variants (CNVs) in 59 genes, and genomic signatures including MSI and TMB. RNA sequencing with hybrid capture detects fusions and splice variants in 55 genes. Variant annotation is performed using the GenomeOncology Precision Oncology Platform (GenomeOncology, Cleveland, OH, United States). Only genomic alterations annotated as known pathogenic were analyzed in the current study.

### 2.3 *NTRK* fusion analysis and classification

*NTRK* fusions were called and assembled using the Manta fusion caller (Chen et al., 2016), and candidate fusions were annotated as [5′ gene]-[3′ gene] when ≥5 unique candidate gene fusion reads were detected (Conroy et al., 2021). Detected fusions were labeled oncogenic or likely oncogenic based on published *NTRK* fusion oncogenicity classification guidelines outlined by the ClinGen *NTRK* Fusions Somatic Cancer Variant Curation Expert Panel (Saliba et al., 2022). Based on these guidelines, *NTRK* fusions were classified as oncogenic if (Amatu et al., 2019) the *NTRK* gene component was the 3′ partner of the fusion and contained the TKD region and (Cocco et al., 2018) the 5′ gene component was a known fusion partner of *NTRK* genes. Known fusion partners included canonical partners or partners with clinical evidence of validity. Canonical fusion partners were defined as those reported in Catalogue Of Somatic Mutations In Cancer (COSMIC) (Sondka et al., 2024) and GENIE (AACR Project GENIE Consortium, 2017) databases or >3 studies in the scientific literature as curated by Salida et al., 2022 (Saliba et al., 2022). Partners with clinical evidence of validity were those identified in clinical studies that supported the FDA approvals of larotrectinib or entrectinib (Hong et al., 2020; Doebele et al., 2020; Drilon et al., 2017). *NTRK* fusions were classified as likely oncogenic if (Amatu et al., 2019) the *NTRK* gene component was the 3′ partner of the fusion and contained the TKD region and (Cocco et al., 2018) the 5′ gene component was a novel fusion partner where the reading frame was preserved (Saliba et al., 2022). An *NTRK* fusion that did not meet the oncogenic or likely oncogenic classification criteria was labeled as a variant of unknown significance (VUS).

### 2.4 Immunohistochemical studies

PD-L1 protein expression on the surface of tumor cells was measured by Dako PD-L1 IHC 22C3 pharmDx (Agilent, Santa Clara, CA, United States). A board-certified anatomical pathologist scored cell surface PD-L1 expression according to published guidelines as tumor proportion score (TPS) or combined positive score (CPS) depending on the tumor type (Patel and Kurzrock, 2015). TPS represents the percentage of tumor cells with positive PD-L1 out of all viable tumor cells. CPS represents the ratio of total PD-L1 positive cells (tumor and non-tumor cells) in a tumor specimen relative to all viable tumor cells. Both are scored numerically with values from 0 to 100.

### 2.5 Statistical analysis

Statistical analysis and plot generation were performed in R v 4.4.2 (https://www.r-project.org/). Basic plotting was performed using the ggplot2 v 3.5.1 (https://cran.r-project.org/web/packages/ggplot2) and ggpubr v 0.6.0 (https://cran.r-project.org/web/packages/ggpubr). Circos plots were generated using the R package circlize v 0.4.16 (https://cran.r-project.org/web/packages/circlize). The Wilcoxon rank-sum test was used to test for differences in TMB and PD-L1 IHC between *NTRK-*positive and negative tumors. TMB was given a pseudo-count of 1 and then log-transformed before statistical testing.

## 3 Results

### 3.1 *NTRK* fusions are present across solid tumor types and histologies

**FIGURE 1:**
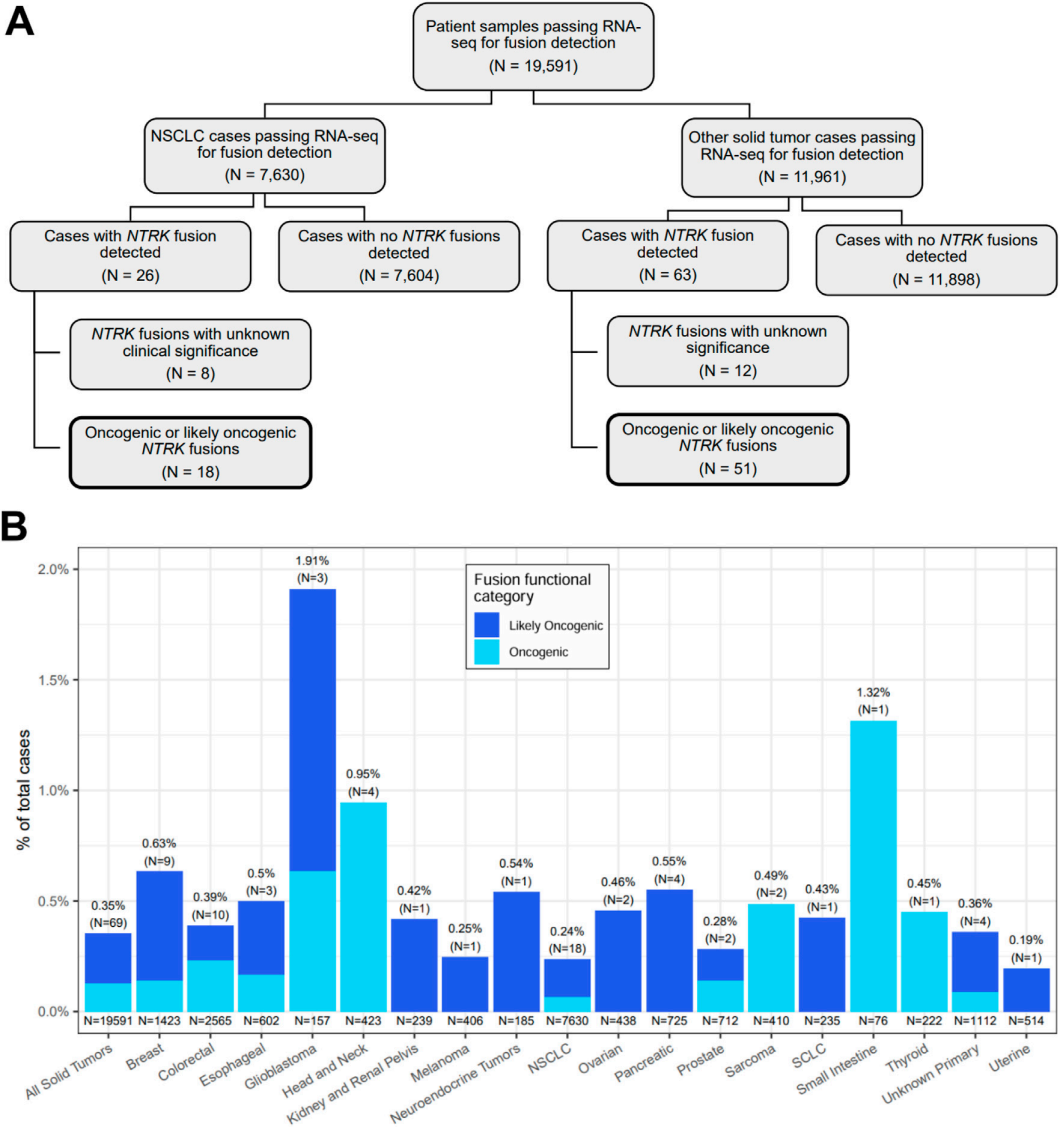
Overview of *NTRK* fusion case selection and detection. **(A)** A flow chart showing the breakdown of *NTRK* fusion cases detected out of 19,591 patient tumor samples tested with and passing the RNA-seq fusion detection component of the TruSight Oncology 500 assay. The remaining figures in the current study focused on cases where oncogenic or likely oncogenic *NTRK* fusions were detected (boxes with bold borders). **(B)** Distributions of oncogenic and likely oncogenic *NTRK* fusions stratified by cancer type. Each bar represents the prevalence of cases with *NTRK* fusions estimated to be oncogenic or likely oncogenic out of the total number of cases that passed RNA-seq for fusion detection. The N in parentheses represents the number of cases with an oncogenic or likely oncogenic *NTRK* fusion. The N under each bar represents the total number of cases included in a group. Each bar is colored by the fraction of cases with *NTRK* fusions predicted to be likely oncogenic or oncogenic via manual data inspection by two independent investigators. NSCLC, non-small cell lung cancer; SCLC, small cell lung cancer.

We retrospectively analyzed data from 19,591 FFPE samples encompassing 35 solid tumor types submitted for CGP as part of routine clinical care (Supplementary Table S1). The largest tumor type group was NSCLC making up more than a third of cases included in the analysis (39%) (Figure 1A; Table 1). Overall, 73 oncogenic or likely oncogenic *NTRK* fusions were identified in 69 unique tumor specimens across 19 tumor types for a total cohort prevalence of 0.35% (Figure 1B, first column; Supplementary Table S2). The prevalence of *NTRK* fusions in NSCLC was below that of the total cohort prevalence (0.24%) and above that in other solid tumor types (0.43%) (Table 1). Tumor types with the highest *NTRK* fusion prevalence included glioblastoma (1.91%, N = 3 out of 157), small intestine (1.32%, N = 1 out of 76), and head and neck (0.95%, N = 4 out of 423) tumors with other solid tumor types ranging from 0.19% (uterine, N = 1 out of 514) to 0.63% (breast, N = 9 out of 1,423) (Figure 1B). The median age of patients with *NTRK* fusions was 66 years (range 26–89 years) with a slight overrepresentation of females in NSCLC (56%) and other solid tumors (61%) (Table 1; Supplementary Table S2). For patients with staging information, almost all were advanced stage (86%) (Table 1; Supplementary Table S2).

**TABLE 1:** Patient characteristics.

	NSCLC (N = 7,630)	Other solid tumors (N = 11,901)
Oncogenic or likely oncogenic *NTRK* fusion prevalence, N (%)	18 (0.24%)	51 (0.43%)
Age (years)		
Median	65.5	66
Range	27–85	26–89
Sex, N (%)		
Male	8 (44%)	20 (39%)
Female	10 (56%)	31 (61%)
Stage, N (%)		
≤III	1 (5%)	3 (6%)
IV	5 (28%)	20 (39%)
Unknown	12 (67%)	28 (55%)

The prevalence of oncogenic or likely oncogenic *NTRK* fusions, is based on the total case N listed at the top of each column. Frequencies for sex and stage are based on the number of oncogenic or likely oncogenic *NTRK*, fusion cases; NSCLC, non-small cell lung cancer.

**FIGURE 2:**
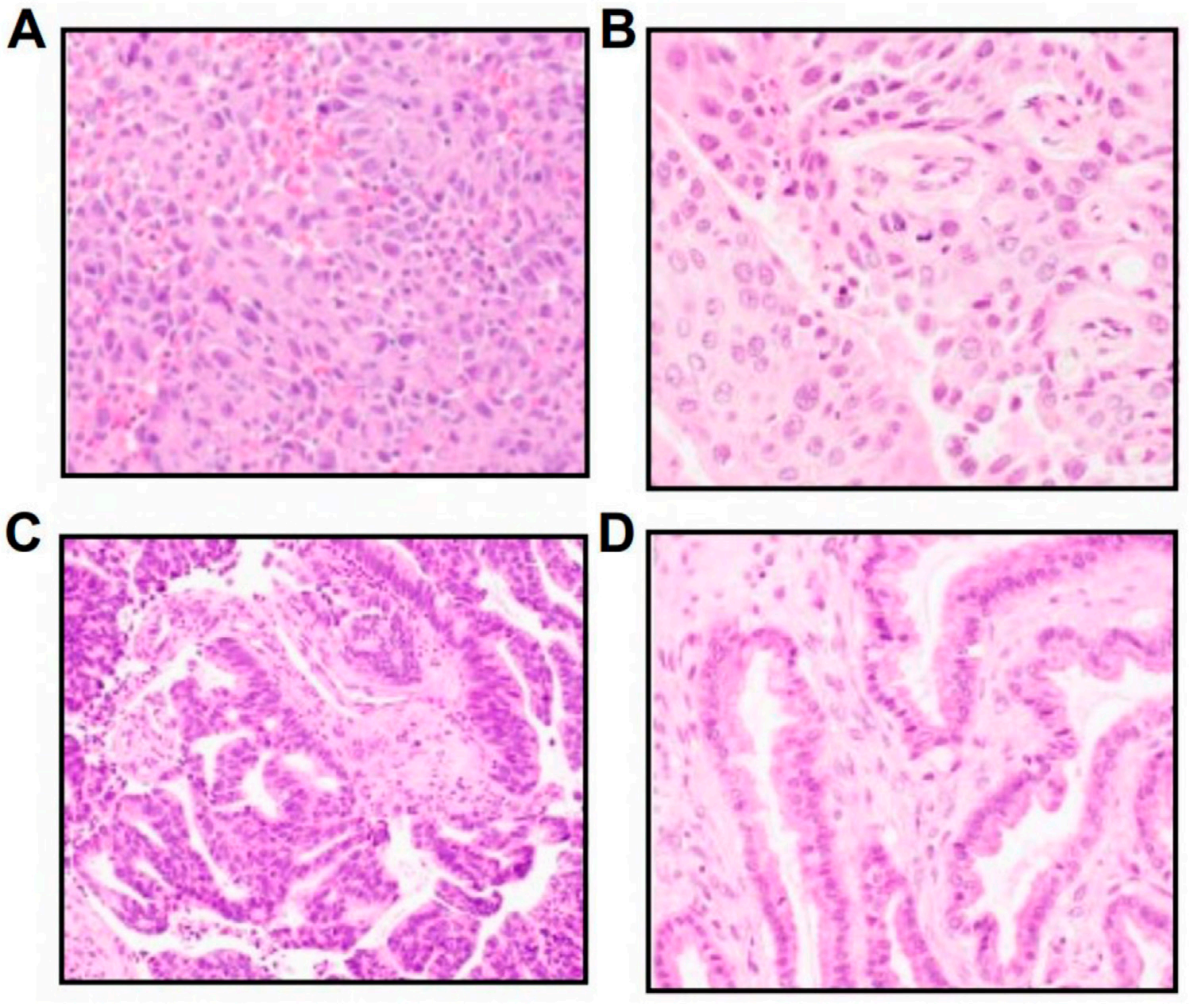
Hematoxylin and eosin images of diverse *NTRK-*positive NSCLC histologies, including **(A)** a poorly differentiated adenocarcinoma with a *RABGAP1L*-*NTRK1* fusion, **(B)** a lung adenocarcinoma with a *TP53*-*NTRK1* fusion, **(C)** a lung adenocarcinoma with enteric differentiation with a *PRKACA*-*NTRK2* fusion, and **(D)** an ovarian carcinoma with a *KANK*-*NTRK3* fusion.

*NTRK* fusions in NSCLC tumors were identified across NSCLC histologic types, including poorly differentiated adenocarcinoma (Figure 2A), lung adenocarcinoma (Figure 2B), and lung adenocarcinoma with enteric differentiation (Figure 2C). For comparison, Figure 2D highlights an ovarian carcinoma with a *KANK-NTRK3* fusion.

### 3.2 Genomic structure of *NTRK* fusions

**FIGURE 3:**
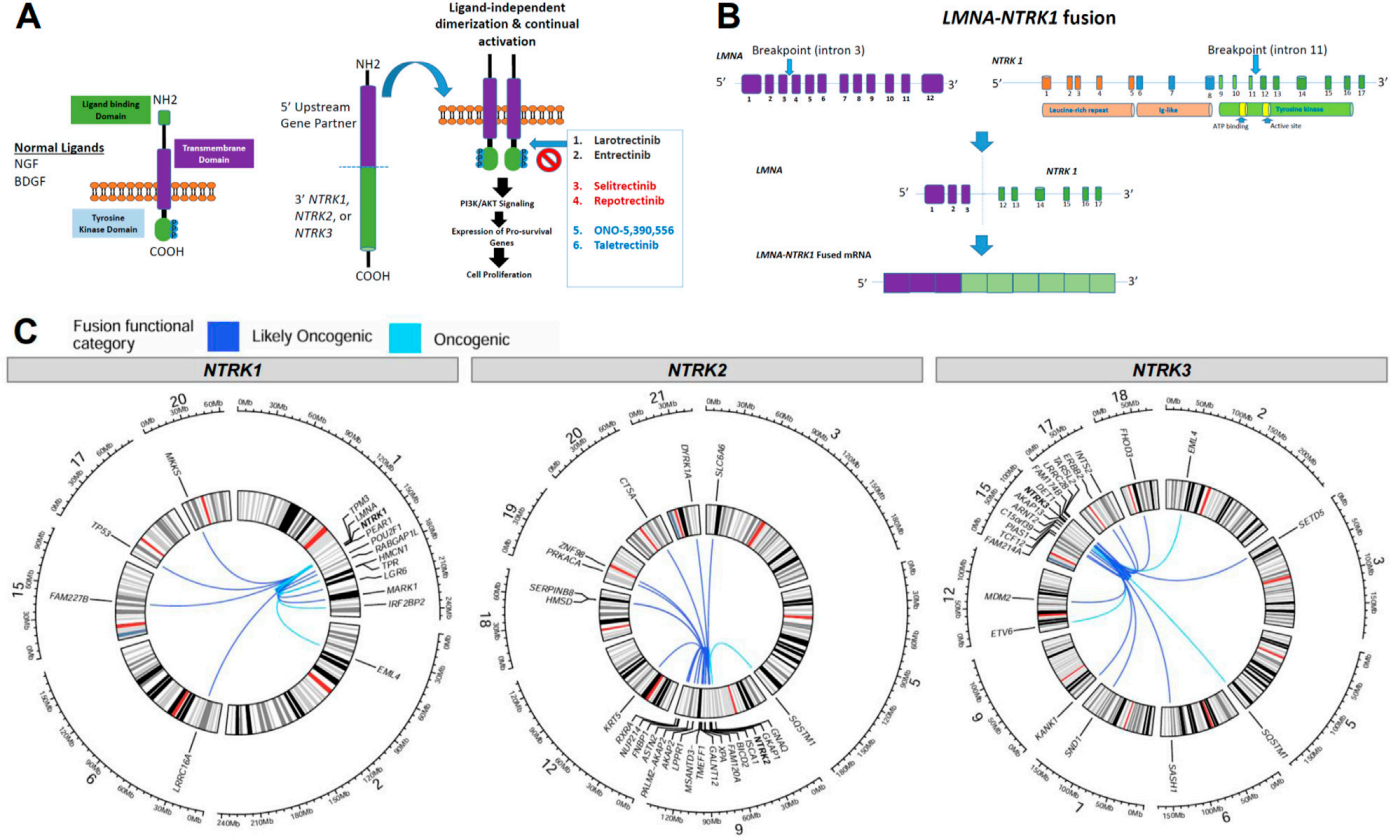
Overview of *NTRK* fusion mechanisms and fusion partners. **(A)** Schematic of the normal neurotrophic tropomyosin receptor kinase (NTRK) structure and oncogenic NTRK fusion structure with downstream pathways and TRK inhibitor therapy options, with next-generation drugs in red and blue. **(B)** An example of breakpoint locations in an *NTRK* fusion (denoted with arrows) in an *LMNA*-*NTRK1* fusion identified in this analysis, resulted in fused mRNA. **(C)** Circos plots demonstrating the locations of *NTRK* genes and fusion partners for oncogenic or likely oncogenic fusions identified in the present study. Lines connect the 5′ and 3′ genes in each gene fusion identified. The color of the lines denotes if a fusion was inferred to be oncogenic or likely oncogenic.

Fusion of the *NTRK* 3′ TKD with a 5′ upstream gene results in a chimeric oncoprotein having ligand-independent constitutive TRK kinase activity (Figure 3A). There are currently multiple generations of *NTRK*-targeted therapies, some approved and others in development, that bind the TRK domain and block downstream growth signaling pathways (Figure 3A). A specific example of a fusion and its structure that was detected in the present study is shown in Figure 3B, where we detail the fusion locations for the known *LMNA-NTRK1* fusion, with breakpoints in intron 3 (*LMNA*) and intron 11 (*NTRK1*), and the resulting *LMNA-NTRK1* fused mRNA.

We identified other, diverse partner genes for oncogenic and likely oncogenic *NTRK* fusions across all tumor types including intra and inter-chromosomal rearrangements with intra-chromosomal fusions identified more frequently (Figure 3C). For *NTRK1-*detected fusions, 10 included partners within chromosome 1 (e.g., *RABGAP1L, PEAR1, LMNA, HMCN1*) while 5 inter-chromosomal rearrangements were identified with genes in chromosomes 2 (*EML4*), 6 (*LRRC16A*), 15 (*FAM227B*), 17 (*TP53*), and 20 (*MKKS*) (Figure 3C, first panel). *NTRK2-*detected fusions had the highest number of unique fusion partners, 15 included partners within chromosome 9 (e.g., *GNAQ*) while 9 inter-chromosomal rearrangements were identified with genes in chromosomes 3 (*SLAC6A6*), 5 (*SQSTM1*), 12 (*KRT5*), 18 (*HMSD*, *SERPINB8*), 19 (*PRKACA*, *ZNF98*), 20 (*CTSA*), and 21 (*DYRK1A*) (Figure 3C, second panel). For *NTRK3*-detected fusions, 10 included partners within chromosome 15 (e.g., *ARNT2*, *AKAP13*) while 11 inter-chromosomal rearrangements were identified with genes in chromosomes 2 (*EML4*), 3 (*SETD5*), 5 (*SQSTM1*), 6 (*SASH1*), 7 (*SND1)*, 9 (*KANK1*), 12 (*ETV6*, *MDM2*), 17 (*ERBB2*, *INTS2*), and 18 (*FHOD3*) (Figure 3C, third panel). Most *NTRK* fusions were detected in only one tumor specimen, though some recurrent fusions were noted with *ETV6*, *TPM3*, *LMNA*, *EML4*, *TPR*, *PEAR1*, *IRF2BP2*, and *KANK1* fusion partners (Table 2; Supplementary Table S2).

**TABLE 2:** Oncogenic or likely oncogenic *NTRK* fusions identified in more than one tumor specimen.

Fusion	N	Fusion %	Cohort %	Functional prediction	Cancer types
*ETV6*-*NTRK3*	5	7.25%	0.025%	Oncogenic	Head and neck (4), Thyroid (1)
*TPM3*-*NTRK1*	3	4.35%	0.015%	Oncogenic	Breast (1), Small intestine (1), Unknown primary (1)
*LMNA*-*NTRK1*	3	4.35%	0.015%	Oncogenic	Colorectal (2), NSCLC (1)
*EML4*-*NTRK3*	2	2.9%	0.01%	Oncogenic	Colorectal (1), Sarcoma (1)
*TPR*-*NTRK1*	2	2.9%	0.01%	Oncogenic	Colorectal (2)
*PEAR1*-*NTRK1*	2	2.9%	0.01%	Oncogenic	Prostate (1), Sarcoma (1)
*IRF2BP2*-*NTRK1*	2	2.9%	0.01%	Oncogenic	Breast (1), NSCLC (1)
*KANK1*-*NTRK3*	2	2.9%	0.01%	Likely oncogenic	Ovarian (2)

N, number of tumor specimens fusion was detected in; Fusion %, proportion of tumor specimens with fusion out of all tumor specimens with oncogenic or likely oncogenic *NTRK* fusions (N = 69); Cohort %, proportion of tumor specimens with fusion out of all tumor specimens analyzed (N = 19,591); Cancer types, the cancer type of each tumor specimen where fusions were detected and the corresponding N (in parentheses) of tumor specimens detected in.

**FIGURE 4:**
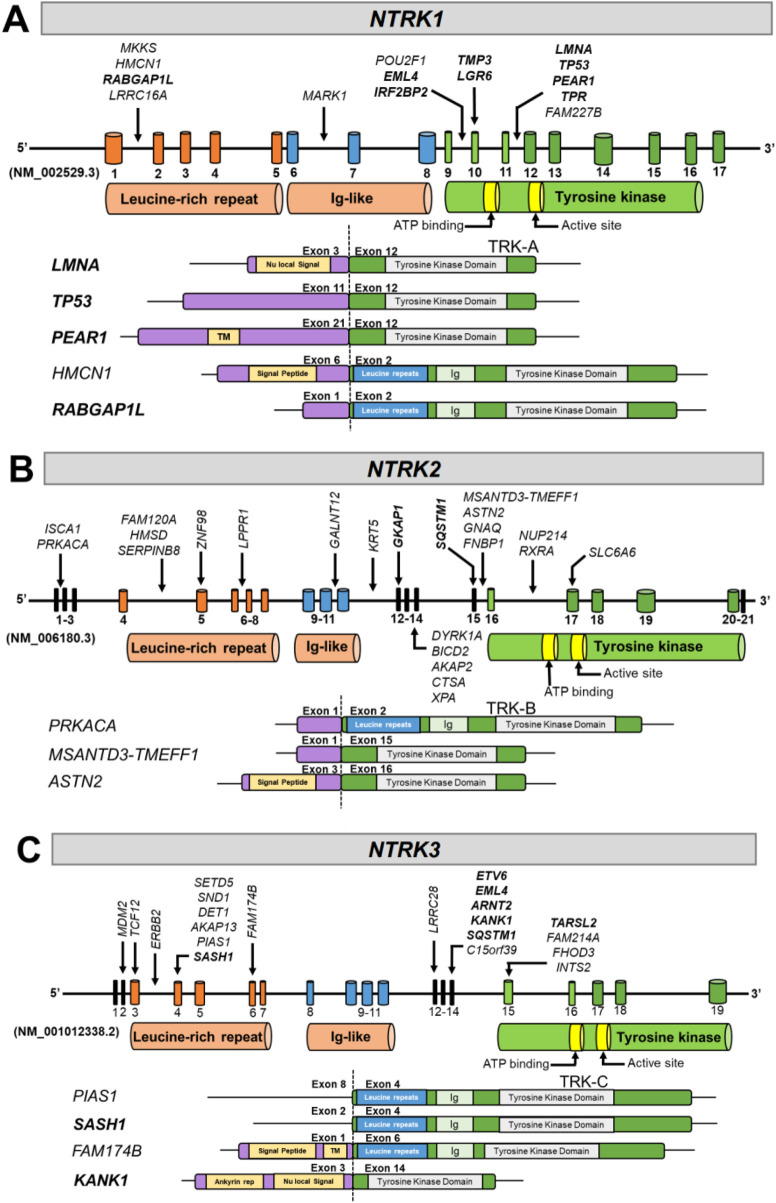
Diagrams of *NTRK* fusions identified in the present study. The resulting exonic structure, including retained exons and functional domains for each partner gene and *NTRK* gene, are represented for **(A)** *NTRK1*, **(B)** *NTRK2*, and **(C)** *NTRK3*. Arrows next to gene partner names denote breakpoints involved in each fusion. Known fusion partners have been bolded. TM, transmembrane; Ig, immunoglobulin.

We identified fusion breakpoint locations in introns 1, 6, 9, 11, and exon 10 in *NTRK1* (Figure 4A; Supplementary Table S2). In *NTRK2,* breakpoints were found more ubiquitously across the *NTRK2* gene including introns 1, 4, 6, 10, 11, 15, 16, and in exons 5, 12, 15, and 17 (Figure 4B; Supplementary Table S2). In *NTRK3*, we identified focalized breakpoints associated with 4–6 gene partners in exons 4, 14, and 15 while other breakpoints were associated with fewer gene partners including intron 3 and exons 2, 3, 6, and 12 (Figure 4C; Supplementary Table S2). We identified 41 novel fusion partners across the *NTRK* genes including *HMCN, ASTN2, MSANTD3-TMEFF1, PRKACA, FAM174B, PIAS1,* and others. We identified fewer known fusion partners (N = 18), which suggests the potential for further investigation into novel fusion partners to increase this number and bring more clinical evidence. Detected known fusion partners included *LMNA, PEAR1, RABGAP1L, TP53, EML4, TMP3, LGR6, TPR,* and *IRF2BP2* for *NTRK1* (Figure 4A), *GKAP1* and *SQSTM1* for *NTRK2* (Figure 4B), and *KANK1, SASH1, ETV6, EML4, ARNT2, SQSTM1,* and *TARSL2* for *NTRK3* (Figure 4C).

The TRK TKD is encoded by different exons in each gene *NTRK* gene. The TKD is encoded by exons 9-17 for *NTRK1*, 16-20 in *NTRK2*, and 15-19 in *NTRK3* (Figure 4). The TKD was present in all gene fusions identified with *NTRK* as the 3′ fusion partner (Figures 4A–C). Transmembrane domains were present in the partner genes *PEAR1* and *FAM174B* (Figures 4A,C), and 6 of 12 *NTRK* fusions contained the transmembrane domain in the TRK protein; however, a transmembrane domain is not required for a fusion to be functional.

### 3.3 Genomic driver alterations that co-occur with *NTRK* fusions

**FIGURE 5:**
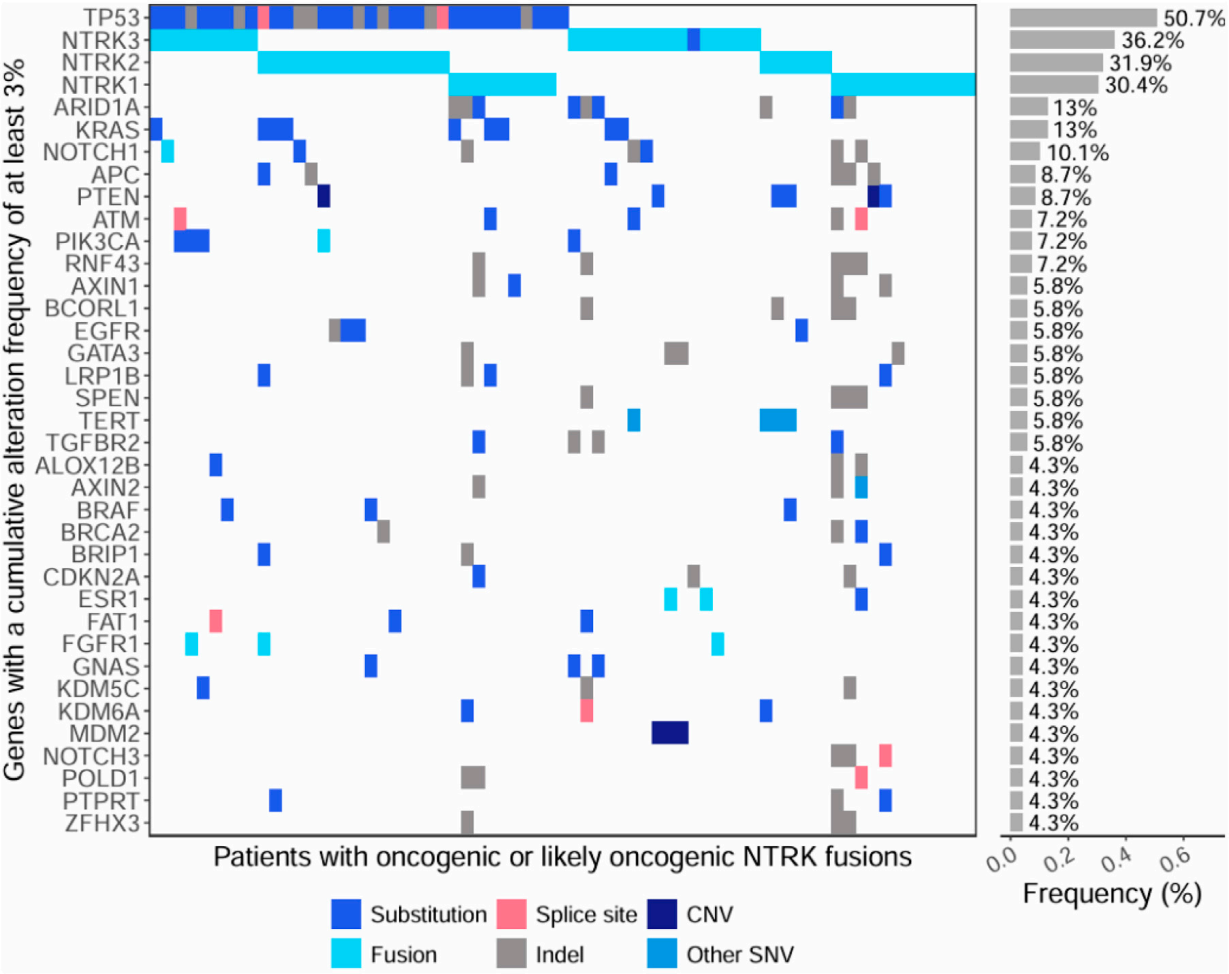
Oncoprint of co-occurring mutations within genes with a cumulative alteration frequency of at least 3%. The first section of the plot shows individual mutations detected within tumors of patients with oncogenic or likely oncogenic NTRK fusions (x-axis). The second section of the plot shows the cumulative alteration frequency of each gene (y-axis). Colors represent the type of genomic alteration identified in the genes.

We evaluated co-occurring genomic alterations across all samples with an oncogenic or likely oncogenic *NTRK* fusion (Figure 5; Supplementary Table S3). The most common co-occurring alterations were small SNVs, or insertions/deletions found in 86% (N = 59) of oncogenic or likely oncogenic *NTRK*-positive tumors. CNVs and non-*NTRK* fusions co-occurred at similar frequencies in 29% (N = 20) and 26% (N = 18) of *NTRK*-positive tumors, respectively (Supplementary Table S3). The gene most commonly co-mutated was *TP53*, which was altered in 50.7% of cases, while other commonly co-mutated genes included *ARID1A* (13%), *KRAS* (13%), and *NOTCH1* (10.1%) (Figure 5; Supplementary Table S3). Co-occurring CNVs typically involved amplification of *MDM2* or loss of *PTEN* (∼4% of *NTRK*-positive tumors respectively) (Figure 5; Supplementary Table S3). Most NTRK fusions were mutually exclusive from other genomic driver alterations, however, almost a third of tumor specimens (29%) contained at least one co-occurring genomic driver (Supplementary Table S3). In NSCLC, co-occurring oncogenic driver mutations were identified in 61% of cases (N = 11 out of 18) including *ALK*, *KRAS, BRAF,* and *EGFR* (Supplementary Table S3). A breakdown of co-occurring alteration frequencies in patient tumors with oncogenic and likely oncogenic *NTRK* fusions and *NTRK* fusions of unknown significance, along with AMP/CAP/ASCO Tier associations, can be found in Supplementary Table S4.

### 3.4 Immunotherapy biomarkers: TMB, MSI, and PD-L1 IHC

**FIGURE 6:**
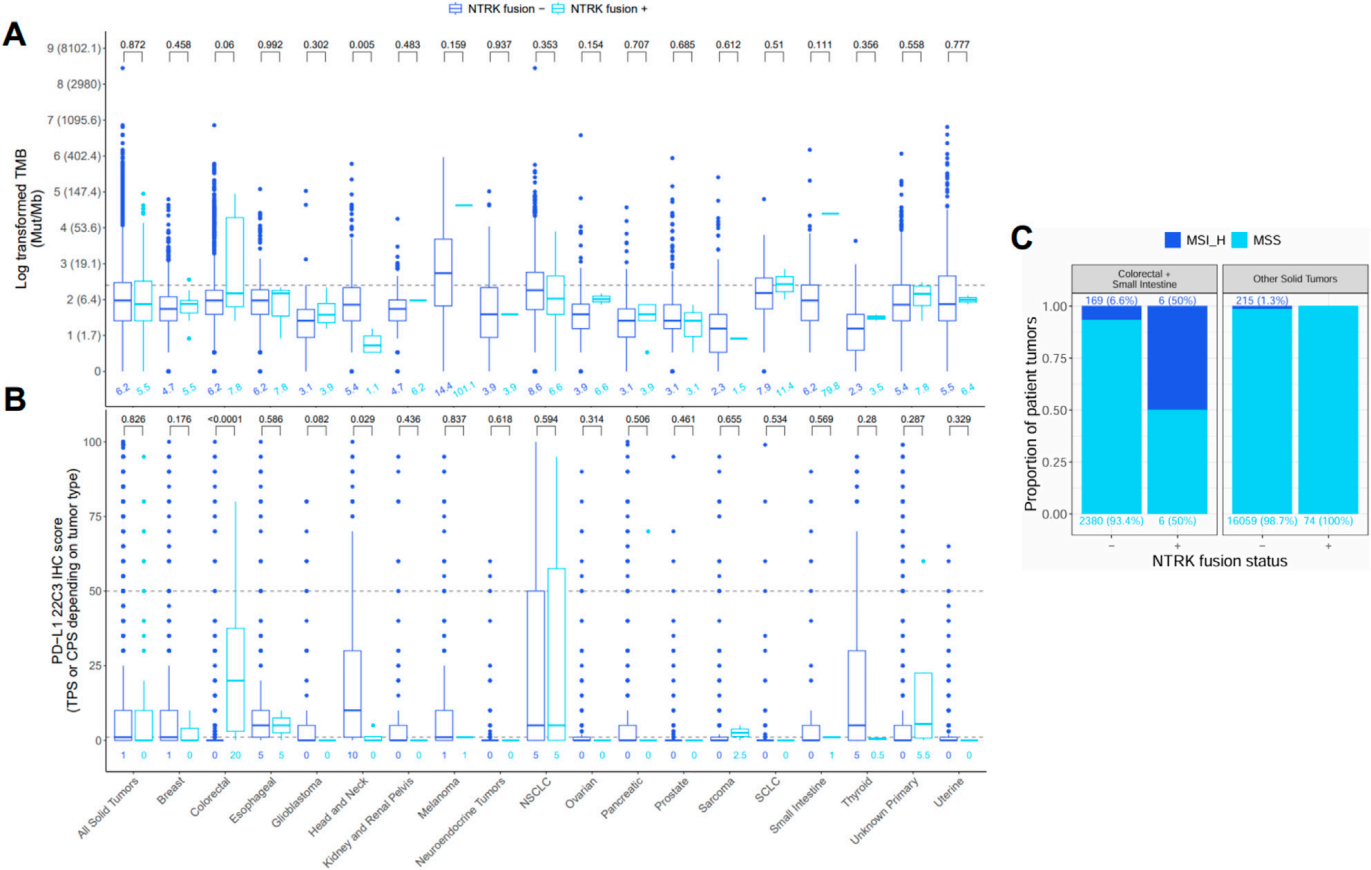
Distribution of **(A)** tumor mutational burden (TMB), **(B)** PD-L1 22C3 immunohistochemistry (IHC), and **(C)** microsatellite instability (MSI) in tumors negative (−) or positive (+) for oncogenic or likely oncogenic *NTRK* fusions. The bottom, middle, and top horizontal boundaries of each box in the box plots represent the first, second (median), and third quartiles of the data for a particular age group. The lines extending from the two ends of each box represent 1.5x outside the interquartile range. Points beyond the lines are considered outliers. Values above each pair of box plots represent the uncorrected P-values from testing differences between *NTRK*–or + tumors using the Wilcoxon rank-sum test. Values below each box plot represent the median value for a group. The dashed line represents the threshold for high (≥10) and low (<10) values for TMB and the high (≥50), low (1–49), and negative (<1) values for PD-L1 IHC. PD-L1 IHC was calculated using the tumor proportion score (TPS) or combined positive score (CPS) depending on tumor type. Values for TMB were log-transformed for testing and plotting, but the corresponding untransformed values are provided in parentheses next to each log-transformed value on the y-axis. Bar plots of MSI represent the proportion of cases that were MSI high or microsatellite stable (MSS) in NTRK - or + tumors. Differences in MSI status were not tested due to low sample size in the MSI-high (MSI_H) groups.

We next sought to characterize distributional differences in immunotherapy biomarkers (TMB, PD-L1 IHC, MSI) between tumors harboring oncogenic or likely oncogenic *NTRK* fusions and *NTRK*-negative tumors (Figure 6). We found TMB and PD-L1 IHC scores were significantly lower in *NTRK*-positive head and neck tumors compared to *NTRK*-negative (1.1 vs. 5.4 mutations/Mb, 0 vs. 10 CPS, P < 0.03) (Figures 6A,B). An opposite trend was observed in CRC tumors where *NTRK*-positive tumors trended toward higher TMB scores (7.8 vs. 6.2 mutations/Mb, P = 0.06) and had significantly higher PD-L1 IHC scores (20 vs. 0 CPS, P < 0.0001) (Figures 6A,B). No significant differences in TMB or PD-L1 IHC were observed when analyzing all solid tumors at once (P > 0.8) (Figures 6A,B). MSI-H was seen at a higher frequency in *NTRK-*positive tumors in CRC and small intestine cases (50% vs. 6.6%) while MSI-H cases were only observed in *NTRK-*negative cases in other solid tumors (Figure 6C).

## 4 Discussion

*NTRK* gene fusions are oncogenic drivers found in diverse pediatric and adult tumors. Intra- and inter-chromosomal rearrangements involving the 3′ regions of *NTRK* genes and the 5′ region of a partner gene can produce a constitutively active TRK fusion protein that drives uncontrolled downstream signaling (Penault-Llorca et al., 2019). Though these gene fusions are rare, they are clinically important targets due to high response rates to targeted therapy and durable responses in patients with TRK fusion-driven advanced and metastatic tumors (Hong et al., 2020; Demetri et al., 2022). It is recommended that all patients with TRK fusion-positive tumors be identified so they can receive approved targeted therapies. In our analysis, 19,531 FFPE solid tumor samples underwent successful RNA hybrid-capture-based NGS and functional characterization of detected *NTRK* fusions, revealing 73 oncogenic or likely oncogenic *NTRK* fusions identified in 69 (0.35%) adult patients (Figure 1; Supplementary Table S2). Among these fusions, 41 novel 5′ partners were identified (Figure 4; Supplementary Table S2).

Though there are several techniques available to identify *NTRK* gene fusions, the wide range of gene partners and the presence of large intronic regions where breakpoints often occur can make identification of these fusions challenging (Marchio et al., 2019). Techniques such as IHC and fluorescence *in situ* hybridization (FISH) are often readily available; however, these methods can produce high rates of false negatives and false positives. Additionally, they do not identify the fusion partner as they only interrogate one target at a time. Reverse transcriptase polymerase chain reaction (RT-PCR) has the advantage of high sensitivity and specificity; however, target sequences must be known ahead of time, which decreases the likelihood of detection of novel fusion partners. Though clinical guidelines acknowledge multiple techniques for *NTRK* gene fusion detection, NGS is the recommended method (Ettinger et al., 2022).

The advantages of using NGS for gene fusion detection include the ability to evaluate multiple actionable targets at once and the potential for detecting novel fusion partners (Marchio et al., 2019). Several commercially available DNA-based hybrid-capture NGS assays exist that sequence full exons or hotspot regions and a select number of introns for detecting common rearrangement and fusion breakpoints (Marchio et al., 2019). Though many breakpoints occur within introns, the number of intronic regions and complete coverage of select introns in targeted panels is often limited to avoid compromising the sequencing capacity of desired exons in key genes of interest (Solomon et al., 2019). Therefore, adequate sequencing of all introns with a DNA-only hybrid capture approach is not feasible when large or complex intronic regions are present. This is especially true for *NTRK* genes that contain large introns that cannot be included in a targeted DNA-only NGS approach. These panels are often designed to sequence a limited number of select introns within *NTRK1* and *NTRK2*. With this approach, breakpoints occurring in introns that are not sequenced in these targeted panels will be missed. Additionally, it is not feasible to sequence the large *NTRK3* introns with targeted DNA panels. These panels are restricted to identifying only *NTRK3* gene fusions with its most common partner, *ETV6*, based on select *ETV6* introns. Because of these limitations, DNA-only targeted NGS platforms will not identify all *NTRK* gene fusions.

RNA-based NGS assays for gene fusion detection eliminate the need for sequencing introns, since they are spliced out in mRNA, and can target known fusion exons within multiple oncogenes. In addition, the detection of RNA fusions indicates successful transcription of the gene fusion and provides insight into possible translation to a functional oncogenic fusion protein. The advantages of using PCR amplicon-based RNA panels for fusion detection include shorter and simpler workflows and compatibility with lower quantities of RNA; however, this technique is limited to a small number of gene targets and detection of novel fusion partners is not possible with classical amplicon approaches. Hybrid capture-based RNA panels typically require high quantities of RNA with more complex and longer workflows but also capture large numbers of gene targets and novel fusions with increased sensitivity and specificity as only one fusion partner needs to be known (Solomon et al., 2019; Singh, 2022; Bruno and Fontanini, 2020).

We utilized an RNA hybrid capture-based NGS approach designed to detect fusions and splice variants in 55 genes including *NTRK1*, *NTRK2,* and *NTRK3* fusions, resulting in a detection rate of 0.35% across a real-world, pan-tumor cohort. The prevalence of *NTRK* fusions in this study was higher than in previous studies using DNA hybrid capture NGS with or without RNA sequencing using targeted multiplex PCR for fusion detection (Westphalen et al., 2021; Rosen et al., 2020). We also used a stringent approach to characterize each *NTRK* fusion as functionally oncogenic or likely oncogenic (Saliba et al., 2022). This resulted in 28 additional *NTRK* fusions detected in this study being classified as VUS and not carried forward in downstream characterizations (Supplementary Table S2). Of the 41 novel fusion partners, 51% (N = 21) were *NTRK2* fusions with the majority having intronic breakpoints (Figure 4B). Detection of novel *NTRK2* fusions is limited using DNA-only hybrid capture approaches due to limited intronic baiting, which would miss most of the novel *NTRK2* fusions identified in this analysis (Westphalen et al., 2021). An oncogenic, canonical *GNAQ-NTRK2* fusion identified in a patient with esophageal adenocarcinoma patient would have likely been missed as breakpoints within both genes were in intronic regions (intron 1 of *GNAQ*, intron 15 of *NTRK2*) that are not typically covered in DNA-only hybrid capture panels. Considering this patient did not have any other actionable co-mutations (*CHEK1* deletion (S343fs) and *TP53* substitution (R248Q), Supplementary Table S3), the identification of this *GNAQ-NTRK2* fusion would be critical for treatment decisions. Additionally, novel *NTRK3* fusions would not have been detected by DNA NGS targeted panels if breakpoints occurred in *NTRK3* introns and did not have *ETV6* as the 5′ fusion partner. The identification of these 41 novel *NTRK* fusion partners in our analysis provides additional evidence that RNA hybrid-capture NGS testing is a superior method for *NTRK* fusion detection over DNA NGS targeted panels and speaks to the need for a well-designed RNA NGS panel to capture all potential *NTRK* fusions.

We assessed TMB and MSI genomic signatures by DNA NGS and PD-L1 expression by IHC in *NTRK* fusion-positive and negative patient tumors. Consistent with a previous report, TMB and PD-L1 expression were found to be significantly lower in *NTRK* fusion-positive head and neck cancer specimens compared to fusion-negative cases (Figures 6A,B), suggesting these *NTRK* fusions are the dominant targetable oncogenic drivers (Xu et al., 2021). DNA NGS was also used to detect co-occurring genomic alterations. Of the 12 *NTRK* fusion-positive CRC and small intestine cases, 50% (N = 6) were MSI-H (Figure 6C) and 92% (N = 11) were *KRAS*, *NRAS*, and *BRAF* wild-type, consistent with published data (Cocco et al., 2019b). One *NTRK* fusion-positive CRC patient’s tumor was MSI-H, TMB-H (91.6 mut/Mb) and PD-L1 IHC positive (CPS score = 80%) with a *POLD1* co-mutation, and another case with metastatic small intestine cancer was also MSI-H and TMB-H (80 mut/Mb) with a *POLD1* co-mutation (Supplementary Tables S2, S3). This suggests these patients are more likely to benefit from immune checkpoint inhibitor (ICI) therapy. ICIs such as pembrolizumab are effective treatment options for patients with MSI-H or TMB-H metastatic CRC (Benson et al., 2021; Andre et al., 2020; Samstein et al., 2019). However, there is currently no evidence to suggest the timing of immunotherapy treatment for NTRK-positive CRC patients. Of the 18 NTRK fusion-positive patients with NSCLC, 6 had PD-L1 IHC expression with TPS ≥1–49%, and 6 had TPS ≥50% for consideration of pembrolizumab (Figure 1B; Supplementary Table S2).

Clinical guidelines emphasize testing for actionable biomarkers (including *ALK, BRAF, EGFR, MET* exon 14 skipping*, NTRK, RET,* and *ROS*) should be performed before administering first-line ICIs due to decreased efficacy when tumors harbor co-occurring driver mutations (Ettinger et al., 2022; Mazieres et al., 2019). Two *NTRK* fusion-positive NSCLC patients had co-occurring *KRAS* G12C driver mutations (Supplementary Table S3). Four *NTRK* fusion-positive patients had co-occurring *EGFR* driver mutations (3 with *EGFR* L858R and 1 with *EGFR* exon 19 deletion), where first-line immunotherapy may have decreased efficacy (Supplementary Table S3) and could also lead to severe immune-related adverse events when followed by osimertinib (Lee et al., 2017; Schoenfeld et al., 2019). Five of these lung cancer patients had novel *NTRK2* fusions with intronic breakpoints that would likely have been missed if using a DNA NGS targeted panel (Supplementary Table S2), potentially leading to first-line immune checkpoint inhibitor therapy with decreased efficacy in the presence of a co-occurring oncogenic driver.

Of the 10 *NTRK* fusion-positive breast cancer patients, only 1 patient had an additional actionable *PIK3CA* E545K mutation (Supplementary Table S3). A novel *ERBB2-NTRK3* fusion was identified from a breast mammary adenocarcinoma specimen that was also confirmed to have an *ERBB2* copy number gain (estimated to be a 7-fold change) and a co-occurring *TP53* mutation (Supplementary Tables S2, S3). The pathology report confirmed this tumor to be HER2 positive (HER2 IHC 3+). In previous NGS genomic profiling studies of HER2+ breast cancer patients, fusion events are common, with *ERBB2* being the most frequently involved gene (Chen et al., 2020) with the most frequent co-mutations found in *TP53* (Schubert et al., 2023; Wang T. et al., 2022). *ERBB2* fusions most commonly occur in chromosome 17, with the most frequent breakpoint identified within exon 27, corresponding to the chromosome and breakpoint identified in this *ERBB2-NTRK3* fusion (Supplementary Table S2). HER2+ patients with tumors harboring *ERBB2* fusions have been shown to achieve a pathological complete response with chemotherapy plus trastuzumab, indicating these fusions do not lead to resistance to therapy (Lesurf et al., 2017). This novel *ERBB2*-*NTRK3* fusion provides an additional targeted therapy option after progression on available anti-HER2 agents.

Additional co-occurring actionable genomic alterations were identified in 5 *NTRK* fusion-positive patients: CRC (*CCDC6-RET* fusion), pancreatic (*ITSN2-ALK* fusion), prostate (*PTEN* loss, *TBL1XR1-PIK3CA* fusion), ovarian (PIK3CA mutation) (Supplementary Table S3). A patient originally diagnosed with uterine endometrioid adenocarcinoma had additional clinically relevant *PIK3CA* and *TP53* mutations that further characterize this patient as having pleomorphic rhabdomyosarcoma (Pinto et al., 2018). In summary, most *NTRK* fusion-positive patients did not have an actionable co-occurring alteration where additional targeted therapies would have been available, highlighting the need for accurate *NTRK* fusion testing.

## 5 Conclusion

*NTRK* fusions are clinically relevant driver alterations across solid tumor types. These fusions are difficult to detect, as breakpoints can occur within introns, and they have a wide range of partner genes. RNA hybrid capture-based sequencing in a real-world standard-of-care setting revealed the highest reportable oncogenic and likely oncogenic *NTRK* fusion prevalence (0.35%) across solid tumors with 41 previously unreported novel *NTRK* fusion partners. These data emphasize the importance of CGP using a well-designed RNA hybrid-capture sequencing assay to identify all *NTRK* fusions across malignancies from a broad range of histologic subtypes for optimal patient treatment management.

## Data Availability

The original contributions presented in the study are included in the article/Supplementary Material, further inquiries can be directed to the corresponding author.

## References

[B1] AACR Project GENIE Consortium, André F. Arnedos M. Baras A. S. Baselga J. Bedard P. L. (2017). AACR Project GENIE: powering precision medicine through an international Consortium. Cancer Discov. 7 (8), 818–831. 10.1158/2159-8290.CD-17-0151 28572459 PMC5611790

[B2] Amatu A. Sartore-Bianchi A. Bencardino K. Pizzutilo E. G. Tosi F. Siena S. (2019). Tropomyosin receptor kinase (TRK) biology and the role of NTRK gene fusions in cancer. Ann. Oncol. 30 (Suppl. 8), viii5–viii15. 10.1093/annonc/mdz383 31738427 PMC6859819

[B3] Andre T. Shiu K. K. Kim T. W. Jensen B. V. Jensen L. H. Punt C. (2020). Pembrolizumab in microsatellite-instability-high advanced colorectal cancer. N. Engl. J. Med. 383 (23), 2207–2218. 10.1056/NEJMoa2017699 33264544

[B4] Bazhenova L. Lokker A. Snider J. Castellanos E. Fisher V. Fellous M. (2021). TRK fusion cancer: patient characteristics and survival analysis in the real-world setting. Target Oncol. 16 (3), 389–399. 10.1007/s11523-021-00815-4 33893941 PMC8105201

[B5] Benson A. B. Venook A. P. Al-Hawary M. M. Arain M. A. Chen Y. J. Ciombor K. K. (2021). Colon cancer, version 2.2021, NCCN clinical practice guidelines in oncology. J. Natl. Compr. Canc Netw. 19 (3), 329–359. 10.6004/jnccn.2021.0012 33724754

[B6] Bruno R. Fontanini G. (2020). Next generation sequencing for gene fusion analysis in lung cancer: a literature review. Diagn. (Basel) 10 (8), 521. 10.3390/diagnostics10080521 PMC7460167 32726941

[B7] Chen B. Zhang G. Wei G. Wang Y. Guo L. Lin J. (2020). Heterogeneity of genomic profile in patients with HER2-positive breast cancer. Endocr. Relat. Cancer 27 (3), 153–162. 10.1530/ERC-19-0414 31905165

[B8] Chen X. Schulz-Trieglaff O. Shaw R. Barnes B. Schlesinger F. Kallberg M. (2016). Manta: rapid detection of structural variants and indels for germline and cancer sequencing applications. Bioinformatics 32 (8), 1220–1222. 10.1093/bioinformatics/btv710 26647377

[B9] Cocco E. Benhamida J. Middha S. Zehir A. Mullaney K. Shia J. (2019b). Colorectal carcinomas containing hypermethylated MLH1 promoter and wild-type BRAF/KRAS are enriched for targetable kinase fusions. Cancer Res. 79 (6), 1047–1053. 10.1158/0008-5472.CAN-18-3126 30643016 PMC6420871

[B10] Cocco E. Scaltriti M. Drilon A. (2018). NTRK fusion-positive cancers and TRK inhibitor therapy. Nat. Rev. Clin. Oncol. 15 (12), 731–747. 10.1038/s41571-018-0113-0 30333516 PMC6419506

[B11] Cocco E. Schram A. M. Kulick A. Misale S. Won H. H. Yaeger R. (2019a). Resistance to TRK inhibition mediated by convergent MAPK pathway activation. Nat. Med. 25 (9), 1422–1427. 10.1038/s41591-019-0542-z 31406350 PMC6736691

[B12] Conroy J. M. Pabla S. Glenn S. T. Seager R. J. Van Roey E. Gao S. (2021). A scalable high-throughput targeted next-generation sequencing assay for comprehensive genomic profiling of solid tumors. PLoS One 16 (12), e0260089. 10.1371/journal.pone.0260089 34855780 PMC8639101

[B13] Demetri G. D. De Braud F. Drilon A. Siena S. Patel M. R. Cho B. C. (2022). Updated integrated analysis of the efficacy and safety of entrectinib in patients with NTRK fusion-positive solid tumors. Clin. Cancer Res. 28 (7), 1302–1312. 10.1158/1078-0432.CCR-21-3597 35144967 PMC9365368

[B14] Doebele R. C. Drilon A. Paz-Ares L. Siena S. Shaw A. T. Farago A. F. (2020). Entrectinib in patients with advanced or metastatic NTRK fusion-positive solid tumours: integrated analysis of three phase 1-2 trials. Lancet Oncol. 21 (2), 271–282. 10.1016/S1470-2045(19)30691-6 31838007 PMC7461630

[B15] Drilon A. (2019). TRK inhibitors in TRK fusion-positive cancers. Ann. Oncol. 30 (Suppl. 8), viii23–viii30. 10.1093/annonc/mdz282 32223935

[B16] Drilon A. Siena S. Ou S. I. Patel M. Ahn M. J. Lee J. (2017). Safety and antitumor activity of the multitargeted pan-TRK, ROS1, and ALK inhibitor entrectinib: combined results from two phase I trials (ALKA-372-001 and STARTRK-1). Cancer Discov. 7 (4), 400–409. 10.1158/2159-8290.CD-16-1237 28183697 PMC5380583

[B17] Ettinger D. S. Wood D. E. Aisner D. L. Akerley W. Bauman J. R. Bharat A. (2022). Non-small cell lung cancer, version 3.2022, NCCN clinical practice guidelines in oncology. J. Natl. Compr. Canc Netw. 20 (5), 497–530. 10.6004/jnccn.2022.0025 35545176

[B18] Gatalica Z. Xiu J. Swensen J. Vranic S. (2019). Molecular characterization of cancers with NTRK gene fusions. Mod. Pathol. 32 (1), 147–153. 10.1038/s41379-018-0118-3 30171197

[B19] Hong D. S. DuBois S. G. Kummar S. Farago A. F. Albert C. M. Rohrberg K. S. (2020). Larotrectinib in patients with TRK fusion-positive solid tumours: a pooled analysis of three phase 1/2 clinical trials. Lancet Oncol. 21 (4), 531–540. 10.1016/S1470-2045(19)30856-3 32105622 PMC7497841

[B20] Klink A. J. Kavati A. Gassama A. T. Kozlek T. Gajra A. Antoine R. (2022). Timing of NTRK gene fusion testing and treatment modifications following TRK fusion status among US oncologists treating TRK fusion cancer. Target Oncol. 17 (3), 321–328. 10.1007/s11523-022-00887-w 35716252 PMC9217884

[B21] Kummar S. Lassen U. N. (2018). TRK inhibition: a new tumor-agnostic treatment strategy. Target Oncol. 13 (5), 545–556. 10.1007/s11523-018-0590-1 30276762

[B22] Lee C. K. Man J. Lord S. Links M. Gebski V. Mok T. (2017). Checkpoint inhibitors in metastatic EGFR-mutated non-small cell lung cancer-A meta-analysis. J. Thorac. Oncol. 12 (2), 403–407. 10.1016/j.jtho.2016.10.007 27765535

[B23] Lesurf R. Griffith O. L. Griffith M. Hundal J. Trani L. Watson M. A. (2017). Genomic characterization of HER2-positive breast cancer and response to neoadjuvant trastuzumab and chemotherapy-results from the ACOSOG Z1041 (Alliance) trial. Ann. Oncol. 28 (5), 1070–1077. 10.1093/annonc/mdx048 28453704 PMC5790063

[B24] Marchio C. Scaltriti M. Ladanyi M. Iafrate A. J. Bibeau F. Dietel M. (2019). ESMO recommendations on the standard methods to detect NTRK fusions in daily practice and clinical research. Ann. Oncol. 30 (9), 1417–1427. 10.1093/annonc/mdz204 31268127

[B25] Mazieres J. Drilon A. Lusque A. Mhanna L. Cortot A. B. Mezquita L. (2019). Immune checkpoint inhibitors for patients with advanced lung cancer and oncogenic driver alterations: results from the IMMUNOTARGET registry. Ann. Oncol. 30 (8), 1321–1328. 10.1093/annonc/mdz167 31125062 PMC7389252

[B26] Okamura R. Boichard A. Kato S. Sicklick J. K. Bazhenova L. Kurzrock R. (2018). Analysis of NTRK alterations in pan-cancer adult and pediatric malignancies: implications for NTRK-targeted therapeutics. JCO Precis. Oncol. 2018, 1–20. 10.1200/PO.18.00183 PMC6329466 30637364

[B27] Patel S. P. Kurzrock R. (2015). PD-L1 expression as a predictive biomarker in cancer immunotherapy. Mol. Cancer Ther. 14 (4), 847–856. 10.1158/1535-7163.MCT-14-0983 25695955

[B28] Penault-Llorca F. Rudzinski E. R. Sepulveda A. R. (2019). Testing algorithm for identification of patients with TRK fusion cancer. J. Clin. Pathol. 72 (7), 460–467. 10.1136/jclinpath-2018-205679 31072837 PMC6589488

[B29] Pinto A. Kahn R. M. Rosenberg A. E. Slomovitz B. Quick C. M. Whisman M. K. (2018). Uterine rhabdomyosarcoma in adults. Hum. Pathol. 74, 122–128. 10.1016/j.humpath.2018.01.007 29320751

[B30] Rosen E. Y. Goldman D. A. Hechtman J. F. Benayed R. Schram A. M. Cocco E. (2020). TRK fusions are enriched in cancers with uncommon histologies and the absence of canonical driver mutations. Clin. Cancer Res. 26 (7), 1624–1632. 10.1158/1078-0432.CCR-19-3165 31871300 PMC7124988

[B31] Saliba J. Church A. J. Rao S. Danos A. Furtado L. V. Laetsch T. (2022). Standardized evidence-based approach for assessment of oncogenic and clinical significance of NTRK fusions. Cancer Genet. 264-265, 50–59. 10.1016/j.cancergen.2022.03.001 35366592 PMC9252326

[B32] Samstein R. M. Lee C. H. Shoushtari A. N. Hellmann M. D. Shen R. Janjigian Y. Y. (2019). Tumor mutational load predicts survival after immunotherapy across multiple cancer types. Nat. Genet. 51 (2), 202–206. 10.1038/s41588-018-0312-8 30643254 PMC6365097

[B33] Schoenfeld A. J. Arbour K. C. Rizvi H. Iqbal A. N. Gadgeel S. M. Girshman J. (2019). Severe immune-related adverse events are common with sequential PD-(L)1 blockade and osimertinib. Ann. Oncol. 30 (5), 839–844. 10.1093/annonc/mdz077 30847464 PMC7360149

[B34] Schubert L. Elliott A. Le A. T. Estrada-Bernal A. Doebele R. C. Lou E. (2023). ERBB family fusions are recurrent and actionable oncogenic targets across cancer types. Front. Oncol. 13, 1115405. 10.3389/fonc.2023.1115405 37168365 PMC10164992

[B35] Singh R. R. (2022). Target enrichment approaches for next-generation sequencing applications in oncology. Diagn. (Basel) 12 (7), 1539. 10.3390/diagnostics12071539 PMC9318977 35885445

[B36] Solomon B. J. Drilon A. Lin J. J. Bazhenova L. Goto K. De Langen J. (2023). 1372P Repotrectinib in patients (pts) with NTRK fusion-positive (NTRK+) advanced solid tumors, including NSCLC: update from the phase I/II TRIDENT-1 trial. Ann. Oncol. 34, S787–S788. 10.1016/j.annonc.2023.09.2405

[B37] Solomon J. P. Benayed R. Hechtman J. F. Ladanyi M. (2019). Identifying patients with NTRK fusion cancer. Ann. Oncol. 30 (Suppl. 8), viii16–viii22. viii16-viii22. 10.1093/annonc/mdz384 32223934

[B38] Sondka Z. Dhir N. B. Carvalho-Silva D. Jupe S. Madhumita M. L. K. (2024). COSMIC: a curated database of somatic variants and clinical data for cancer. Nucleic Acids Res. 52 (D1), D1210–D1217. 10.1093/nar/gkad986 38183204 PMC10767972

[B39] Wang H. Li Z. W. Ou Q. Wu X. Nagasaka M. Shao Y. (2022a). NTRK fusion positive colorectal cancer is a unique subset of CRC with high TMB and microsatellite instability. Cancer Med. 11 (13), 2541–2549. 10.1002/cam4.4561 35506567 PMC9249987

[B40] Wang T. Wei L. Lu Q. Shao Y. You S. Yin J. C. (2022b). Landscape of potentially targetable receptor tyrosine kinase fusions in diverse cancers by DNA-based profiling. NPJ Precis. Oncol. 6 (1), 84. 10.1038/s41698-022-00325-0 36369474 PMC9652465

[B41] Westphalen C. B. Krebs M. G. Le Tourneau C. Sokol E. S. Maund S. L. Wilson T. R. (2021). Genomic context of NTRK1/2/3 fusion-positive tumours from a large real-world population. NPJ Precis. Oncol. 5 (1), 69. 10.1038/s41698-021-00206-y 34285332 PMC8292342

[B42] Wong D. Yip S. Sorensen P. H. (2020). Methods for identifying patients with tropomyosin receptor kinase (TRK) fusion cancer. Pathol. Oncol. Res. 26 (3), 1385–1399. 10.1007/s12253-019-00685-2 31256325 PMC7297824

[B43] Xu J. Wang R. Wang T. Wang T. Gu D. He Y. (2021). Targeted DNA profiling and the prevalence of NTRK aberrations in Chinese patients with head and neck cancer. Oral Oncol. 119, 105369. 10.1016/j.oraloncology.2021.105369 34098386

