# Supplementary material for: RNA hybrid-capture next-generation sequencing has high sensitivity in identifying known and less characterized oncogenic and likely oncogenic NTRK fusions in a real-world standard-of-care setting
Source: Front Genet. 2025 May 2;16:1550706. doi: 10.3389/fgene.2025.1550706 (PMC12081445; doi:10.3389/fgene.2025.1550706)
Supplement: Supplementary file 2 [file DataSheet1.pdf]

```
# get R environment ready
library(readxl)
library(plyr)
library(ggplot2)
library(ggpubr)
library(circlize)
memoSort <- function(M) {
  geneOrder <- sort(
    rowSums(!is.na(M) & M != "" & M > 0 & M != FALSE),
    decreasing = TRUE, index.return = TRUE
  )$ix
  scoreCol <- function(x) {
    score <- 0
    for (i in 1:length(x)) {
      if (!is.na(x[i]) & x[i] != "" & x[i] > 0 & x[i] != FALSE) {
        score <- score + 2^(length(x) - i)
      }
    }
    return(score)
  }
  scores <- apply(M[geneOrder, ], 2, scoreCol)
  sampleOrder <- sort(scores, decreasing = TRUE, index.return = TRUE)$ix
  return(M[geneOrder, sampleOrder])
}
labcorp.col <- c(
  "#3a5ce9", "#4cd5f7", "#f7758c", "#918f90",
  "#1a2188", "#2998e3", "#6c2fac", "#b4f6f5"
)

# import data
ntrk.list <- data.frame(read_xlsx("Data/NTRK_fusion_list.xlsx", sheet = 1))
patient.df <- data.frame(read_xlsx(
  "Data/All_OI_de_id_patient_data.xlsx",
  sheet = 1,
  na = c("NULL", "NA", "N/A")
))
variant.df <- data.frame(read_xlsx(
  "Data/All_OI_de_id_genomic_results.xlsx",
  sheet = 1,
  na = c("NULL", "NA", "N/A")
))

# differentiate between NSCLC and SCLC
patient.df$omnidisease_fullname[grep("Lung / Malignant Epithelial / Small cell",
patient.df$mcode_path)] <- "SCLC"
patient.df$omnidisease_fullname[
  grepl("Lung / Malignant Epithelial", patient.df$mcode_path) &
    patient.df$omnidisease_fullname != "SCLC"
] <- "NSCLC"

# replace brain and nervous system with glioblastoma
patient.df$omnidisease_fullname[grep("Brain", patient.df$omnidisease_fullname)] <-
"Glioblastoma"

# create variable denoting who had a NTRK fusion and its pathogenic prediction
patient.df$ntrk.fusion <- ifelse(
  patient.df$de_id %in% ntrk.list$de_id[ntrk.list$Functional.prediction ==
"Oncogenic"], "Oncogenic",
  ifelse(
```

```
    patient.df$de_id %in% ntrk.list$de_id[ntrk.list$Functional.prediction == "Likely
Oncogenic"], "Likely Oncogenic",
    ifelse(
      patient.df$de_id %in% ntrk.list$de_id[ntrk.list$Functional.prediction ==
"VUS"], "VUS",
      ifelse(
        patient.df$RNA_Rearrangements == "Pass", "Not Detected", NA
      )
    )
  )
)
patient.df$ntrk.fusion <- factor(patient.df$ntrk.fusion, levels = c("Oncogenic",
"Likely Oncogenic", "VUS", "Not Detected"))

# recode SNV type variable to make more simple categories and add CNV and Fusion
entries
variant.df$snv_type[grep("^Complex|^Nonstop|^Promoter|Unknown", variant.df$snv_type)]
<- "Other SNV"
variant.df$snv_type[grep("^Deletion|^Insertion", variant.df$snv_type)] <- "Indel"
variant.df$snv_type[grep("^Splice", variant.df$snv_type)] <- "Splice site"
variant.df$snv_type[grep("^Substitution", variant.df$snv_type)] <- "Substitution"
variant.df$snv_type[variant.df$variant_type == "CNV"] <- "CNV"
variant.df$snv_type[variant.df$variant_type == "Fusion"] <- "Fusion"

# plot frequencies of NTRK fusions out of all cases
prop.df <- ddply(
  patient.df, .(omnidisease_fullname, ntrk.fusion), summarize,
  count = length(de_id)
)
prop.df <- prop.df[
  prop.df$omnidisease_fullname %in%
    prop.df$omnidisease_fullname[prop.df$ntrk.fusion != "Not Detected" &
!is.na(prop.df$ntrk.fusion)],
]
for (i in unique(prop.df$omnidisease_fullname)) {
  prop.df$prop[prop.df$omnidisease_fullname == i] <-
    prop.df$count[prop.df$omnidisease_fullname == i] /
      sum(na.omit(patient.df$omnidisease_fullname == i &
patient.df$RNA_Rearrangements == "Pass"))
}
prop.df <- rbind(
  data.frame(
    omnidisease_fullname = "All Solid Tumors",
    ntrk.fusion = levels(patient.df$ntrk.fusion),
    count = as.vector(table(patient.df$ntrk.fusion)),
    prop = as.vector(table(patient.df$ntrk.fusion)) /
sum(na.omit(patient.df$RNA_Rearrangements == "Pass"))
  ),
  prop.df[!is.na(prop.df$ntrk.fusion) &
    prop.df$omnidisease_fullname %in%
      c("All Solid Tumors",
names(table(patient.df$omnidisease_fullname))[table(patient.df$omnidisease_fullname)
> 20]), ]
)
g <- ggplot(
  prop.df[!(prop.df$ntrk.fusion %in% c("VUS", "Not Detected")), ],
  aes(x = gsub(" of the Skin", "", gsub(" Cancer", "", omnidisease_fullname)), y =
prop)
) +
  geom_col(aes(fill = ntrk.fusion)) +
```

```
  geom_text(
    data = ddply(
      prop.df[!(prop.df$ntrk.fusion %in% c("VUS", "Not Detected")), ],
.(omnidisease_fullname), summarize,
      sum.prop = sum(prop), sum.count = sum(count)
    ),
    aes(y = sum.prop, label = paste(round(sum.prop * 100, 2), "%\n(N=", sum.count,
")", sep = "")),
    size = 2.5, vjust = -0.25
  ) +
  geom_text(
    data = ddply(prop.df, .(omnidisease_fullname), summarize, sum.count =
sum(count))[
      ddply(prop.df, .(omnidisease_fullname), summarize, sum.count =
sum(count))$omnidisease_fullname %in%
        prop.df$omnidisease_fullname[!(prop.df$ntrk.fusion %in% c("VUS", "Not
Detected"))],
    ],
    aes(y = 0, label = paste("N=", sum.count, sep = "")),
    size = 2.5, vjust = 1.5
  ) +
  scale_y_continuous(labels = scales::percent, limits = c(0, 0.02)) +
  scale_fill_manual(name = "Fusion functional\ncategory", values = labcorp.col[1:3])
+
  theme_bw() +
  labs(y = "% of total cases", x = "Cancer type") +
  theme(
    text = element_text(size = 10),
    axis.text.x = element_text(angle = 30, hjust = 1)
  )
ggsave("Output/Plots/NTRK_fusion_prevalence.pdf", g, device = "pdf", height = 5,
width = 10)


# create circos plot of NTRK1 fusion partners
bed1 <- data.frame(
  chr.1 = paste(
    "chr",
    sapply(
      strsplit(
        variant.df$variant_mut_hash[
          variant.df$variant %in% ntrk.list$Variant[
            grepl("NTRK1", ntrk.list$Variant) & ntrk.list$Functional.prediction !=
"VUS"
          ]
        ], ":"
      ),
      function(x) {
        as.numeric(substr(x[3], start = 1, stop = 2))
      }
    ),
    sep = ""
  ),
  start.1 = sapply(
    strsplit(
      variant.df$variant_mut_hash[
        variant.df$variant %in% ntrk.list$Variant[
          grepl("NTRK1", ntrk.list$Variant) & ntrk.list$Functional.prediction !=
"VUS"
        ]
```

```
      ], ":"
    ),
    function(x) {
      sapply(
        strsplit(x[3], "\\|"),
        function(x) {
          as.numeric(substr(x[1], start = 3, stop = nchar(x[1])))
        }
      )
    }
  ),
  end.1 = sapply(
    strsplit(
      variant.df$variant_mut_hash[
        variant.df$variant %in% ntrk.list$Variant[
          grepl("NTRK1", ntrk.list$Variant) & ntrk.list$Functional.prediction !=
"VUS"
        ]
      ], ":"
    ),
    function(x) {
      sapply(
        strsplit(x[3], "\\|"),
        function(x) {
          as.numeric(substr(x[1], start = 3, stop = nchar(x[1])))
        }
      )
    }
  ),
  gene.1 = sapply(
    strsplit(
      variant.df$variant_mut_hash[
        variant.df$variant %in% ntrk.list$Variant[
          grepl("NTRK1", ntrk.list$Variant) & ntrk.list$Functional.prediction !=
"VUS"
        ]
      ], ":"
    ),
    function(x) {
      x[1]
    }
  )
)
bed2 <- data.frame(
  chr.2 = paste(
    "chr",
    sapply(
      strsplit(
        variant.df$variant_mut_hash[
          variant.df$variant %in% ntrk.list$Variant[
            grepl("NTRK1", ntrk.list$Variant) & ntrk.list$Functional.prediction !=
"VUS"
          ]
        ], ":"
      ),
      function(x) {
        as.numeric(substr(x[5], start = 1, stop = 2))
      }
    ),
    sep = ""
```

```
  ),
  start.2 = sapply(
    strsplit(
      variant.df$variant_mut_hash[
        variant.df$variant %in% ntrk.list$Variant[
          grepl("NTRK1", ntrk.list$Variant) & ntrk.list$Functional.prediction !=
"VUS"
        ]
      ], ":"
    ),
    function(x) {
      sapply(
        strsplit(x[5], " "),
        function(x) {
          as.numeric(substr(x[1], start = 3, stop = nchar(x[1])))
        }
      )
    }
  ),
  end.2 = sapply(
    strsplit(
      variant.df$variant_mut_hash[
        variant.df$variant %in% ntrk.list$Variant[
          grepl("NTRK1", ntrk.list$Variant) & ntrk.list$Functional.prediction !=
"VUS"
        ]
      ], ":"
    ),
    function(x) {
      sapply(
        strsplit(x[5], " "),
        function(x) {
          as.numeric(substr(x[1], start = 3, stop = nchar(x[1])))
        }
      )
    }
  ),
  gene.2 = sapply(
    strsplit(
      variant.df$variant_mut_hash[
        variant.df$variant %in% ntrk.list$Variant[
          grepl("NTRK1", ntrk.list$Variant) & ntrk.list$Functional.prediction !=
"VUS"
        ]
      ], ":"
    ),
    function(x) {
      sapply(
        strsplit(x[3], "\\|"),
        function(x) {
          x[2]
        }
      )
    }
  )
)
bed <- unique(cbind(bed1, bed2))
bed <- ddply(
  data.frame(bed, gene.combo = gsub("-", "_", paste(bed$gene.1, bed$gene.2, sep =
"_"))), .(gene.combo), summarize,
```

```
  chr.1 = unique(chr.1), start.1 = max(start.1), end.1 = max(start.1), gene.1 =
unique(gene.1),
  chr.2 = unique(chr.2), start.2 = max(start.2), end.2 = max(start.2), gene.2 =
unique(gene.2)
)
label.df <- ddply(
  rbind(
    data.frame(chr = bed$chr.1, start = bed$start.1, end = bed$end.1, gene =
gsub("MSANTD3-", "MSANTD3-\n", bed$gene.1)),
    data.frame(chr = bed$chr.2, start = bed$start.2, end = bed$end.2, gene =
bed$gene.2)
  ),
  .(gene), summarize,
  chr = unique(chr), start = min(start), end = max(end)
)
pdf("Output/Plots/NTRK1_fusion_circos.pdf", height = 5, width = 5)
circos.clear()
circos.par("start.degree" = 90, "gap.degree" = rep(4, length(unique(c(bed$chr.1,
bed$chr.2)))))
circos.initializeWithIdeogram(
  plotType = c("axis", "labels"),
  chromosome.index = paste("chr", sort(as.numeric(gsub("chr", "", unique(c(bed$chr.1,
bed$chr.2))))), sep = "")
)
circos.genomicLabels(
  label.df[, c("chr", "start", "end", "gene")],
  labels.column = 4, side = "outside", cex = 0.5,
  font = ifelse(grepl("NTRK", label.df$gene), 4, 3)
)
circos.genomicIdeogram(track.height = convert_height(7, "mm"))
circos.genomicLink(
  data.frame(chr = bed$chr.1, start = bed$start.1, end = bed$end.1),
  data.frame(chr = bed$chr.2, start = bed$start.2, end = bed$end.2),
  col = ifelse(
    bed$gene.combo %in%
      gsub(
        "-", "_",
        gsub(
          " Fusion", "",
          ntrk.list$Variant[ntrk.list$Functional.prediction == "Oncogenic"]
        )
      ), labcorp.col[2],
    ifelse(
      bed$gene.combo %in%
        gsub(
          "-", "_",
          gsub(
            " Fusion", "",
            ntrk.list$Variant[ntrk.list$Functional.prediction == "Likely Oncogenic"]
          )
        ), labcorp.col[1], "grey"
    )
  ),
  border = NA
)
trash <- dev.off()

# create circos plot of NTRK2 fusion partners
bed1 <- data.frame(
  chr.1 = paste(
```

```
    "chr",
    sapply(
      strsplit(
        variant.df$variant_mut_hash[
          variant.df$variant %in% ntrk.list$Variant[
            grepl("NTRK2", ntrk.list$Variant) & ntrk.list$Functional.prediction !=
"VUS"
          ]
        ], ":"
      ),
      function(x) {
        as.numeric(substr(x[3], start = 1, stop = 2))
      }
    ),
    sep = ""
  ),
  start.1 = sapply(
    strsplit(
      variant.df$variant_mut_hash[
        variant.df$variant %in% ntrk.list$Variant[
          grepl("NTRK2", ntrk.list$Variant) & ntrk.list$Functional.prediction !=
"VUS"
        ]
      ], ":"
    ),
    function(x) {
      sapply(
        strsplit(x[3], "\\|"),
        function(x) {
          as.numeric(substr(x[1], start = 3, stop = nchar(x[1])))
        }
      )
    }
  ),
  end.1 = sapply(
    strsplit(
      variant.df$variant_mut_hash[
        variant.df$variant %in% ntrk.list$Variant[
          grepl("NTRK2", ntrk.list$Variant) & ntrk.list$Functional.prediction !=
"VUS"
        ]
      ], ":"
    ),
    function(x) {
      sapply(
        strsplit(x[3], "\\|"),
        function(x) {
          as.numeric(substr(x[1], start = 3, stop = nchar(x[1])))
        }
      )
    }
  ),
  gene.1 = sapply(
    strsplit(
      variant.df$variant_mut_hash[
        variant.df$variant %in% ntrk.list$Variant[
          grepl("NTRK2", ntrk.list$Variant) & ntrk.list$Functional.prediction !=
"VUS"
        ]
      ], ":"
```

```
    ),
    function(x) {
      x[1]
    }
  )
)
bed2 <- data.frame(
  chr.2 = paste(
    "chr",
    sapply(
      strsplit(
        variant.df$variant_mut_hash[
          variant.df$variant %in% ntrk.list$Variant[
            grepl("NTRK2", ntrk.list$Variant) & ntrk.list$Functional.prediction !=
"VUS"
          ]
        ], ":"
      ),
      function(x) {
        as.numeric(substr(x[5], start = 1, stop = 2))
      }
    ),
    sep = ""
  ),
  start.2 = sapply(
    strsplit(
      variant.df$variant_mut_hash[
        variant.df$variant %in% ntrk.list$Variant[
          grepl("NTRK2", ntrk.list$Variant) & ntrk.list$Functional.prediction !=
"VUS"
        ]
      ], ":"
    ),
    function(x) {
      sapply(
        strsplit(x[5], " "),
        function(x) {
          as.numeric(substr(x[1], start = 3, stop = nchar(x[1])))
        }
      )
    }
  ),
  end.2 = sapply(
    strsplit(
      variant.df$variant_mut_hash[
        variant.df$variant %in% ntrk.list$Variant[
          grepl("NTRK2", ntrk.list$Variant) & ntrk.list$Functional.prediction !=
"VUS"
        ]
      ], ":"
    ),
    function(x) {
      sapply(
        strsplit(x[5], " "),
        function(x) {
          as.numeric(substr(x[1], start = 3, stop = nchar(x[1])))
        }
      )
    }
  ),
```

```
  gene.2 = sapply(
    strsplit(
      variant.df$variant_mut_hash[
        variant.df$variant %in% ntrk.list$Variant[
          grepl("NTRK2", ntrk.list$Variant) & ntrk.list$Functional.prediction !=
"VUS"
        ]
      ], ":"
    ),
    function(x) {
      sapply(
        strsplit(x[3], "\\|"),
        function(x) {
          x[2]
        }
      )
    }
  )
)
bed <- unique(cbind(bed1, bed2))
bed <- ddply(
  data.frame(bed, gene.combo = gsub("-", "_", paste(bed$gene.1, bed$gene.2, sep =
"_"))), .(gene.combo), summarize,
  chr.1 = unique(chr.1), start.1 = max(start.1), end.1 = max(start.1), gene.1 =
unique(gene.1),
  chr.2 = unique(chr.2), start.2 = max(start.2), end.2 = max(start.2), gene.2 =
unique(gene.2)
)
label.df <- ddply(
  rbind(
    data.frame(chr = bed$chr.1, start = bed$start.1, end = bed$end.1, gene =
gsub("MSANTD3-", "MSANTD3-\n", bed$gene.1)),
    data.frame(chr = bed$chr.2, start = bed$start.2, end = bed$end.2, gene =
bed$gene.2)
  ),
  .(gene), summarize,
  chr = unique(chr), start = min(start), end = max(end)
)
pdf("Output/Plots/NTRK2_fusion_circos.pdf", height = 5, width = 5)
circos.clear()
circos.par("start.degree" = 90, "gap.degree" = rep(4, length(unique(c(bed$chr.1,
bed$chr.2)))))
circos.initializeWithIdeogram(
  plotType = c("axis", "labels"),
  chromosome.index = paste("chr", sort(as.numeric(gsub("chr", "", unique(c(bed$chr.1,
bed$chr.2))))), sep = "")
)
circos.genomicLabels(
  label.df[, c("chr", "start", "end", "gene")],
  labels.column = 4, side = "outside", cex = 0.5,
  font = ifelse(grepl("NTRK", label.df$gene), 4, 3)
)
circos.genomicIdeogram(track.height = convert_height(7, "mm"))
circos.genomicLink(
  data.frame(chr = bed$chr.1, start = bed$start.1, end = bed$end.1),
  data.frame(chr = bed$chr.2, start = bed$start.2, end = bed$end.2),
  col = ifelse(
    bed$gene.combo %in%
      gsub(
        "-", "_",
```

```
        gsub(
          " Fusion", "",
          ntrk.list$Variant[ntrk.list$Functional.prediction == "Oncogenic"]
        )
      ), labcorp.col[2],
    ifelse(
      bed$gene.combo %in%
        gsub(
          "-", "_",
          gsub(
            " Fusion", "",
            ntrk.list$Variant[ntrk.list$Functional.prediction == "Likely Oncogenic"]
          )
        ), labcorp.col[1], "grey"
    )
  ),
  border = NA
)
trash <- dev.off()

# create circos plot of NTRK3 fusion partners
bed1 <- data.frame(
  chr.1 = paste(
    "chr",
    sapply(
      strsplit(
        variant.df$variant_mut_hash[
          variant.df$variant %in% ntrk.list$Variant[
            grepl("NTRK3", ntrk.list$Variant) & ntrk.list$Functional.prediction !=
"VUS"
          ]
        ], ":"
      ),
      function(x) {
        as.numeric(substr(x[3], start = 1, stop = 2))
      }
    ),
    sep = ""
  ),
  start.1 = sapply(
    strsplit(
      variant.df$variant_mut_hash[
        variant.df$variant %in% ntrk.list$Variant[
          grepl("NTRK3", ntrk.list$Variant) & ntrk.list$Functional.prediction !=
"VUS"
        ]
      ], ":"
    ),
    function(x) {
      sapply(
        strsplit(x[3], "\\|"),
        function(x) {
          as.numeric(substr(x[1], start = 3, stop = nchar(x[1])))
        }
      )
    }
  ),
  end.1 = sapply(
    strsplit(
      variant.df$variant_mut_hash[
```

```
        variant.df$variant %in% ntrk.list$Variant[
          grepl("NTRK3", ntrk.list$Variant) & ntrk.list$Functional.prediction !=
"VUS"
        ]
      ], ":"
    ),
    function(x) {
      sapply(
        strsplit(x[3], "\\|"),
        function(x) {
          as.numeric(substr(x[1], start = 3, stop = nchar(x[1])))
        }
      )
    }
  ),
  gene.1 = sapply(
    strsplit(
      variant.df$variant_mut_hash[
        variant.df$variant %in% ntrk.list$Variant[
          grepl("NTRK3", ntrk.list$Variant) & ntrk.list$Functional.prediction !=
"VUS"
        ]
      ], ":"
    ),
    function(x) {
      x[1]
    }
  )
)
bed2 <- data.frame(
  chr.2 = paste(
    "chr",
    sapply(
      strsplit(
        variant.df$variant_mut_hash[
          variant.df$variant %in% ntrk.list$Variant[
            grepl("NTRK3", ntrk.list$Variant) & ntrk.list$Functional.prediction !=
"VUS"
          ]
        ], ":"
      ),
      function(x) {
        as.numeric(substr(x[5], start = 1, stop = 2))
      }
    ),
    sep = ""
  ),
  start.2 = sapply(
    strsplit(
      variant.df$variant_mut_hash[
        variant.df$variant %in% ntrk.list$Variant[
          grepl("NTRK3", ntrk.list$Variant) & ntrk.list$Functional.prediction !=
"VUS"
        ]
      ], ":"
    ),
    function(x) {
      sapply(
        strsplit(x[5], " "),
        function(x) {
```

```
          as.numeric(substr(x[1], start = 3, stop = nchar(x[1])))
        }
      )
    }
  ),
  end.2 = sapply(
    strsplit(
      variant.df$variant_mut_hash[
        variant.df$variant %in% ntrk.list$Variant[
          grepl("NTRK3", ntrk.list$Variant) & ntrk.list$Functional.prediction !=
"VUS"
        ]
      ], ":"
    ),
    function(x) {
      sapply(
        strsplit(x[5], " "),
        function(x) {
          as.numeric(substr(x[1], start = 3, stop = nchar(x[1])))
        }
      )
    }
  ),
  gene.2 = sapply(
    strsplit(
      variant.df$variant_mut_hash[
        variant.df$variant %in% ntrk.list$Variant[
          grepl("NTRK3", ntrk.list$Variant) & ntrk.list$Functional.prediction !=
"VUS"
        ]
      ], ":"
    ),
    function(x) {
      sapply(
        strsplit(x[3], "\\|"),
        function(x) {
          x[2]
        }
      )
    }
  )
)
bed <- unique(cbind(bed1, bed2))
bed <- ddply(
  data.frame(bed, gene.combo = gsub("-", "_", paste(bed$gene.1, bed$gene.2, sep =
"_"))), .(gene.combo), summarize,
  chr.1 = unique(chr.1), start.1 = max(start.1), end.1 = max(start.1), gene.1 =
unique(gene.1),
  chr.2 = unique(chr.2), start.2 = max(start.2), end.2 = max(start.2), gene.2 =
unique(gene.2)
)
label.df <- ddply(
  rbind(
    data.frame(chr = bed$chr.1, start = bed$start.1, end = bed$end.1, gene =
gsub("MSANTD3-", "MSANTD3-\n", bed$gene.1)),
    data.frame(chr = bed$chr.2, start = bed$start.2, end = bed$end.2, gene =
bed$gene.2)
  ),
  .(gene), summarize,
  chr = unique(chr), start = min(start), end = max(end)
```

```
)
pdf("Output/Plots/NTRK3_fusion_circos.pdf", height = 5, width = 5)
circos.clear()
circos.par("start.degree" = 90, "gap.degree" = rep(4, length(unique(c(bed$chr.1,
bed$chr.2)))))
circos.initializeWithIdeogram(
  plotType = c("axis", "labels"),
  chromosome.index = paste("chr", sort(as.numeric(gsub("chr", "", unique(c(bed$chr.1,
bed$chr.2))))), sep = "")
)
circos.genomicLabels(
  label.df[, c("chr", "start", "end", "gene")],
  labels.column = 4, side = "outside", cex = 0.5,
  font = ifelse(grepl("NTRK", label.df$gene), 4, 3)
)
circos.genomicIdeogram(track.height = convert_height(7, "mm"))
circos.genomicLink(
  data.frame(chr = bed$chr.1, start = bed$start.1, end = bed$end.1),
  data.frame(chr = bed$chr.2, start = bed$start.2, end = bed$end.2),
  col = ifelse(
    bed$gene.combo %in%
      gsub(
        "-", "_",
        gsub(
          " Fusion", "",
          ntrk.list$Variant[ntrk.list$Functional.prediction == "Oncogenic"]
        )
      ), labcorp.col[2],
    ifelse(
      bed$gene.combo %in%
        gsub(
          "-", "_",
          gsub(
            " Fusion", "",
            ntrk.list$Variant[ntrk.list$Functional.prediction == "Likely Oncogenic"]
          )
        ), labcorp.col[1], "grey"
    )
  ),
  border = NA
)
trash <- dev.off()

# create matrix of genes and detected alterations for each patient
alt.matrix <- t(
  reshape2::acast(
    variant.df[variant.df$de_id %in%
      ntrk.list$de_id[ntrk.list$Functional.prediction != "VUS"], ],
    de_id ~ gene,
    value.var = "snv_type",
    fun.aggregate = function(x) {
      ifelse(length(x) == 0, "", x)
    }
  )
)
alt.matrix[alt.matrix == ""] <- NA

# calculate combined frequencies of alterations per gene
gene.freq <- ddply(
  variant.df[
```

```
    variant.df$de_id %in%
      ntrk.list$de_id[ntrk.list$Functional.prediction != "VUS"],
  ],
  .(gene), summarize,
  var_count = length(unique(de_id)),
  var_freq = length(unique(de_id)) /
    length(
      unique(
        variant.df$de_id[
          variant.df$de_id %in%
            ntrk.list$de_id[ntrk.list$Functional.prediction != "VUS"]
        ]
      )
    )
)

# remove genes that have <3% prevalence when rounded using calculated gene
frequencies
alt.matrix <- alt.matrix[gene.freq$gene[gene.freq$var_freq >= 0.03], ]

# sort alteration matrix based on mutual exclusivity of alterations
alt.matrix <- memoSort(alt.matrix)

# create color vector for filling in plots based on alteration type
col <- labcorp.col[1:length(unique(alt.matrix[!is.na(alt.matrix)]))]
names(col) <- unique(alt.matrix[!is.na(alt.matrix)])

# create waterfall plot
g1 <- ggplot(
  reshape2::melt(alt.matrix[rev(rownames(alt.matrix)), ]),
  aes(
    y = Var1,
    x = factor(as.character(Var2), levels = as.character(unique(Var2))),
    fill = value
  )
) +
  geom_tile(color = NA) +
  scale_fill_manual(
    breaks = unique(alt.matrix[!is.na(alt.matrix)]), values = col, na.value = NA,
    labels = unique(alt.matrix[!is.na(alt.matrix)])
  ) +
  theme_bw() +
  theme(
    text = element_text(size = 16),
    axis.text.y = element_text(size = 14),
    axis.text.x = element_blank(),
    axis.ticks.x = element_blank(),
    legend.title = element_blank(),
    legend.position = "bottom",
    panel.border = element_rect(linewidth = 1),
    panel.grid.major = element_blank(), panel.grid.minor = element_blank()
  ) +
  labs(
    y = "Genes with a cumulative alteration frequency of at least 3%",
    x = "Patients with oncogenic or likely oncogenic NTRK fusions",
  )
gene.sub <- gene.freq[gene.freq$var_freq >= 0.03, ]
gene.sub$gene <- factor(gene.sub$gene, levels = rev(rownames(alt.matrix)))
g2 <- ggplot(data = gene.sub, aes(x = gene, y = var_freq)) +
  geom_bar(stat = "identity", alpha = 0.5, width = 0.8) +
```

```
  geom_text(
    aes(label = paste(round(var_freq * 100, 1), "%", sep = "")),
    size = 4.5, hjust = -0.1
  ) +
  scale_y_continuous(
    limits = c(0, max(gene.freq$var_freq[gene.freq$var_freq >= 0.03]) + 0.2)
  ) +
  coord_flip() +
  theme_classic() +
  labs(y = "Frequency (%)", x = "") +
  guides(fill = guide_legend(override.aes = list(shape = NA))) +
  theme(
    text = element_text(size = 16),
    axis.text.x = element_text(angle = 30, hjust = 1),
    axis.title.y = element_blank(),
    axis.text.y = element_blank(),
    axis.ticks.y = element_blank(),
    axis.line.x.bottom = element_line(color = "black"),
    axis.line.y.left = element_blank()
  )
g <- cowplot::plot_grid(
  g1, NULL, g2,
  align = "h", axis = "bt",
  nrow = 1, ncol = 3, rel_widths = c(1, 0, 0.25)
)
ggsave("Output/Plots/NTRK_fusion_waterfall.pdf", g, device = "pdf", width = 10,
height = 8)

# plot distributions of TMB between NTRK fusion and non-NTRK fusion cases
plot.data <- rbind(
  patient.df[
    !is.na(patient.df$ntrk.fusion) &
      patient.df$omnidisease_fullname %in%
        patient.df$omnidisease_fullname[patient.df$de_id %in%
ntrk.list$de_id[!(ntrk.list$Functional.prediction %in% c("VUS", "Not Detected"))]] &
      patient.df$omnidisease_fullname %in%

names(table(patient.df$omnidisease_fullname))[table(patient.df$omnidisease_fullname)
> 20],
  ],
  patient.df[!is.na(patient.df$ntrk.fusion), ]
)
plot.data$omnidisease_fullname[
  (nrow(plot.data) - nrow(patient.df[!is.na(patient.df$ntrk.fusion), ]) +
1):nrow(plot.data)
] <- "All Solid Tumors"
plot.data$ntrk.fusion <- ifelse(
  plot.data$ntrk.fusion != "Not Detected" & !is.na(plot.data$ntrk.fusion), "NTRK
fusion +",
  ifelse(!is.na(plot.data$ntrk.fusion), "NTRK fusion -", NA)
)
summ.stats <- ddply(
  plot.data, .(omnidisease_fullname, ntrk.fusion), summarize,
  TMB = median(na.omit(TMB))
)
g <- ggplot(
  plot.data,
  aes(
    y = log(TMB + 1),
    x = gsub(" of the Skin", "", gsub(" Cancer", "", omnidisease_fullname)),
```

```
    color = ntrk.fusion
  )
) +
  geom_hline(yintercept = log(10 + 1), linetype = "dashed", alpha = 0.5) +
  geom_boxplot(position = position_dodge(0.9)) +
  geom_pwc() +
  geom_text(
    data = summ.stats, aes(y = -0.5, color = ntrk.fusion, label = round(TMB, 1)),
size = 4,
    position = position_dodge(0.9), angle = 30, show.legend = FALSE
  ) +
  scale_y_continuous(
    breaks = seq(0, 9),
    labels = c(
      0,
      sapply(
        seq(1, 9),
        function(x) {
          paste(x, " (", round(exp(x) - 1, 1), ")", sep = "")
        }
      )
    )
  ) +
  scale_color_manual(name = "", values = labcorp.col) +
  coord_cartesian(ylim = c(-0.5, 9), clip = "off") +
  theme_classic() +
  labs(y = "Log transformed TMB\n(Mut/Mb)", x = "") +
  theme(
    text = element_text(size = 16),
    axis.text.x = element_text(angle = 45, hjust = 1),
    axis.title.x = element_blank(),
    axis.title.y = element_text(vjust = 1),
    legend.position = "top"
  )
ggsave("Output/Plots/NTRK_fusion_log_TMB_distribution.pdf", g, device = "pdf", width
= 15, height = 8)

# plot distributions of PD-L1 IHC between NTRK fusion and non-NTRK fusion cases
plot.data <- rbind(
  patient.df[
    !is.na(patient.df$ntrk.fusion) &
      patient.df$omnidisease_fullname %in%
        patient.df$omnidisease_fullname[patient.df$de_id %in%
ntrk.list$de_id[!(ntrk.list$Functional.prediction %in% c("VUS", "Not Detected"))]] &
      patient.df$omnidisease_fullname %in%

names(table(patient.df$omnidisease_fullname))[table(patient.df$omnidisease_fullname)
> 20],
  ],
  patient.df[!is.na(patient.df$ntrk.fusion), ]
)
plot.data$omnidisease_fullname[
  (nrow(plot.data) - nrow(patient.df[!is.na(patient.df$ntrk.fusion), ]) +
1):nrow(plot.data)
] <- "All Solid Tumors"
plot.data$ntrk.fusion <- ifelse(
  plot.data$ntrk.fusion != "Not Detected" & !is.na(plot.data$ntrk.fusion), "NTRK
fusion +",
  ifelse(!is.na(plot.data$ntrk.fusion), "NTRK fusion -", NA)
)
```

```
summ.stats <- ddply(
  plot.data, .(omnidisease_fullname, ntrk.fusion), summarize,
  PD_L1_IHC_22C3_result = median(na.omit(PD_L1_IHC_22C3_result))
)
g <- ggplot(
  plot.data,
  aes(
    y = PD_L1_IHC_22C3_result,
    x = gsub(" of the Skin", "", gsub(" Cancer", "", omnidisease_fullname)),
    color = ntrk.fusion
  )
) +
  geom_hline(yintercept = 1, linetype = "dashed", alpha = 0.5) +
  geom_hline(yintercept = 50, linetype = "dashed", alpha = 0.5) +
  geom_boxplot(position = position_dodge(0.9)) +
  geom_pwc() +
  geom_text(
    data = summ.stats, aes(y = -5, color = ntrk.fusion, label =
round(PD_L1_IHC_22C3_result, 1)), size = 4,
    position = position_dodge(0.9), show.legend = FALSE
  ) +
  scale_color_manual(name = "", values = labcorp.col) +
  theme_classic() +
  labs(y = "PD-L1 22C3 IHC score\n(TPS or CPS depending on tumor type)", x = "") +
  theme(
    text = element_text(size = 16),
    axis.text.x = element_text(angle = 45, hjust = 1),
    axis.title.x = element_blank(),
    axis.title.y = element_text(vjust = 1),
    legend.position = "top"
  )
ggsave("Output/Plots/NTRK_fusion_PD_L1_distribution.pdf", g, device = "pdf", width =
15, height = 8)

# plot distributions of MSI high between NTRK fusion and non-NTRK fusion cases
fish.test <- function(a, b) {
  return(fisher.test(cbind(a, b)))
}
prop.df <- patient.df[!is.na(patient.df$MSI) & patient.df$MSI != "FT", ]
prop.df$ntrk.fusion <- ifelse(
  prop.df$ntrk.fusion != "Not Detected" & !is.na(prop.df$ntrk.fusion), "+",
  ifelse(!is.na(prop.df$ntrk.fusion), "-", NA)
)
prop.df$omnidisease_fullname[
  grep("Colo|Small", prop.df$omnidisease_fullname)
] <- "Colorectal +\nSmall Intestine"
prop.df$omnidisease_fullname[
  grep("Colorectal", prop.df$omnidisease_fullname, invert = TRUE)
] <- "Other Solid Tumors"
prop.df <- ddply(
  prop.df, .(omnidisease_fullname, ntrk.fusion, MSI), summarize,
  count = length(de_id)
)
prop.df <- prop.df[!is.na(prop.df$ntrk.fusion), ]
for (i in unique(prop.df$omnidisease_fullname)) {
  for (j in unique(prop.df$ntrk.fusion)) {
    prop.df$prop[prop.df$omnidisease_fullname == i & prop.df$ntrk.fusion == j] <-
      prop.df$count[prop.df$omnidisease_fullname == i & prop.df$ntrk.fusion == j] /
        sum(na.omit(
          prop.df$count[prop.df$omnidisease_fullname == i & prop.df$ntrk.fusion == j]
```

```
        ))
  }
}
g <- ggplot(prop.df, aes(x = ntrk.fusion, y = prop, fill = MSI)) +
  geom_bar(position = "stack", stat = "identity") +
  facet_grid(. ~ omnidisease_fullname) +
  geom_text(
    aes(
      y = ifelse(MSI == "MSI_H", 1.01, -0.05),
      color = MSI,
      label = paste(count, " (", round(prop, 3) * 100, "%)", sep = "")
    ),
    vjust = 0
  ) +
  scale_fill_manual(name = "", values = labcorp.col) +
  scale_color_manual(values = labcorp.col) +
  theme_bw() +
  guides(color = "none") +
  labs(y = "Proportion of patient tumors", x = "NTRK fusion status") +
  theme(
    text = element_text(size = 16),
    axis.title.y = element_text(vjust = 1),
    legend.position = "top",
    strip.text = element_text(size = 10)
  )
ggsave("Output/Plots/NTRK_fusion_MSI_distribution.pdf", g, device = "pdf", width = 5,
height = 5)
```
